# Current evidence on powered versus manual circular staplers in colorectal surgery: a systematic review and meta-analysis

**DOI:** 10.1007/s00384-025-04807-y

**Published:** 2025-01-15

**Authors:** José Martín-Arévalo, David Moro-Valdezate, Leticia Pérez-Santiago, Fernando López-Mozos, Carlos Javier Peña, Juan Antonio Carbonell Asins, David Casado Rodrigo, Stephanie García-Botello, Claudia Gil-Alfosea, Vicente Pla-Martí

**Affiliations:** 1 Colorectal Surgery Unit, Department of General and Digestive Surgery, Hospital Clínico Universitario de Valencia, Av. Blasco Ibáñez, 17. 46010, Valencia, Spain; 2 Department of Surgery, University of Valencia, Valencia, Spain; 3 Unit of Biostatistics, INCLIVA Biomedical Research Institute, Valencia, Spain

**Keywords:** Two-row manual circular stapler, Powered circular stapler, Anastomotic leak, Anastomotic bleed, Postoperative anastomotic complications

## Abstract

**Purpose:**

This meta-analysis aims to evaluate the efficacy of powered circular staplers (PCS) compared to manual circular staplers (MCS) in reducing anastomotic leakage (AL) and postoperative bleeding (AB) in colorectal surgery.

**Methods:**

Extensive searches were performed in the Embase, PubMed, and SCOPUS electronic bibliographic databases. Most studies were of an observational nature, and only one randomized clinical trial was identified.

**Results:**

Twelve studies met the inclusion criteria for anastomotic leakage and five for anastomotic hemorrhage. The number of patients included for AL analysis was 4524. The leakage rate was 4.6% (208 cases). The number of patients with AB was 2868 with a bleeding rate of 4.99% (143 patients). After identifying outliers and studies with possible selection bias, the odds ratio (OR) for leaks and PCS was 0.38 (95% CI 0.26–0.55), the relative risk was − 0.05 (95% CI − 0.07 to 0.03), and the number needed to treat to prevent one leak was 20. For bleeding, the PCS OR for PCS was 0.20 (95% CI 0.0772–0.5177).

**Conclusion:**

Powered circular staplers could be associated with a significantly lower risk of leakage and anastomotic bleeding than two-row manual circular staplers. Further prospective randomized trials are needed to validate these findings.

**Supplementary Information:**

The online version contains supplementary material available at 10.1007/s00384-025-04807-y.

## Introduction

Colorectal anastomotic leakage remains one of the most significant complications in colorectal surgery, affecting up to 30% of cases and leading to severe physiological and psychological consequences for patients [1–3]. This complication frequently requires additional interventions such as interventional radiology, reoperations, and stoma creation, all of which profoundly impact patient outcomes and increase morbidity. The management of anastomotic dehiscence also places a substantial burden on healthcare systems, leading to an increase in healthcare resource utilization and associated costs [4–10].

Anastomotic leakage results from a multifactorial process involving patient-related factors, surgical technique, and postoperative care. Patient comorbidities, such as poor nutritional status, diabetes, and immunosuppression, are often not modifiable [11], yet prehabilitation programs can have an impact on potentially modifiable risk factors. Therefore, current strategies focus on optimizing the patient’s physiological condition before surgery through prehabilitation protocols [12] and enhancing recovery through multimodal rehabilitation programs like ERAS [13]. Despite these advancements, technical factors during surgery still play a critical role in determining anastomotic outcomes.

In recent years, several technological innovations have been introduced to reduce incidence of anastomotic leakage. Among the most notable are the use of indocyanine green fluorescence imaging for real-time assessment of anastomotic vascularization [14], and the development of new circular staplers designed to improve stapling accuracy and potentially reduce the risk of anastomotic complications. The first of these new circular staplers to appear on the market was the Echelon Powered Circular Stapler (EPCS), which in addition to the powered firing process combines two stapling innovations (3D Stapler design and Gripping Surface Technology) created to improve healing conditions along the anastomotic line [15–17]. Subsequently, a three-row circular stapler was developed based on three circular rows of conventional B-shaped staples varying in height [18, 19].

More recently, outcomes have been published of a second new powered circular stapling device, the Intocare Powered Circular Stapler (ICS) [20]. However, this powered circular stapler shares only the automated firing mechanism and staple design in common with the first powered circular stapler (EPCS) [21].

Since the publication of first meta-analysis about EPCS [22], five new studies have emerged [20, 23–25]. Given the influx of new data, there is a clear need for a more comprehensive and methodologically rigorous meta-analysis to provide a definitive answer regarding the efficacy of powered circular staplers.

Therefore, the objective of this study was to evaluate the outcomes of powered circular staplers (PCS) compared to two-row manual circular staplers (MCS) in colorectal surgery, focusing on their impact on the incidence of anastomotic leaks and postoperative bleeding. By conducting a comprehensive meta-analysis, this study aims to provide a clear and unbiased assessment of the effectiveness of these stapling devices.

## Material and methods

This meta-analysis was registered in PROSPERO (CRD42024531620). The study was conducted following the updated PRISMA guidelines [26] for systematic reviews and meta-analyses (Supplementary material. PRISMA).

### Search strategy

A comprehensive search in the electronic bibliographic databases PubMed, Embase, and SCOPUS was carried out using the following terms to identify articles for meta-analysis: “powered circular stapler,” “circular powered stapler,” “circular” and “powered” and “stapler,” “Echelon” and “circular” and “stapler,” “Echelon” and “powered” and “circular” and “stapler,” “IntoCare OR ICS” and “circular” “stapler.”. No language restrictions or time limits were applied.

### Eligibility criteria

The selection criteria included: comparative studies of the outcomes of PCS versus MCS published in any language in indexed journals, conference abstracts published in index-supported journals, and papers that clearly identified and reported the primary outcome of anastomotic leakage and/or the secondary outcome of anastomotic bleeding.

Anastomotic leakage after anterior resection of colorectal cancer (CRC) was defined as communication between intra- and extraluminal compartments due to a defect in bowel wall integrity at colonic/rectal or colonic/anal anastomoses [27].

Anastomotic bleeding was defined as two or more episodes of rectal bleeding with a concomitant decrease in hemoglobin level requiring endoscopic evaluation [28].

Exclusion criteria included studies that did not meet the inclusion criteria, studies in which the actual number of anastomotic leaks was not specified and animal experimental studies.

### Selection of studies and data extraction

Two authors (VPM and JMA) independently searched the three bibliographic databases used. Each author then carried out a selection of relevant studies based on the PICO’s eligibility criteria [29]. The study population comprised patients aged > 18 years who underwent circular stapler colorectal anastomosis, the intervention included the use of PCS, a comparison between PCS and MCS was performed, and the main outcome was anastomotic leak while the secondary outcome evaluated was anastomotic bleeding.

Studies meeting the selection criteria were then evaluated via title, abstract, and full-text review. Key details from each study were documented in a custom-designed meta-analysis form using Excel 2016. Variables recorded included: authors, year of publication, number of PCS leaks, number of cases without PCS leaks, total number of PCS cases, number of MCS leaks, number of cases without MCS leaks, total number of MCS cases, and pathologies included in the study (mixed or malignant exclusively).

After the selection of studies, the two authors compared their results for the final selection of publications. In cases of uncertainty or disagreement, a third author (DMV) was consulted to resolve the identified discrepancies.

### Risk of bias and quality assessment

All studies were independently evaluated by two authors (JMA and VPM) using the ROBINS I tool [30]. A third author (DMV) confirmed the final determination after discussion.

GRADE methodology was used to assess the overall quality of evidence [31–34]. GRADE classifies evidence or results into one of four levels: high, moderate, low and very low. Risk of bias, inconsistency, inaccuracy, indirect evidence, or a strong likelihood of publication bias are criteria that can decrease confidence in the results by one or two levels depending on the severity of the issue. We also created a GRADE table to summarize the study.

### Assessment of risk of publication bias

In this study, funnel plots were constructed to visualize publication bias, and Egger’s test was employed for further assessment. Additionally, we investigated the potential for p-hacking, which involves the manipulation or selective reporting of data to achieve statistical significance. In the context of meta-analysis, the presence of p-hacking introduces potential bias, compromising the statistical integrity and reliability of the findings. To evaluate the risk of publication bias and p-hacking, we conducted specific analyses, including right-skewness and flatness tests, which allow a rigorous evaluation of potential biases in the reported results.

### Statistical analysis

The association between stapler type and the occurrence of anastomotic complications, such as leakage or bleeding, was evaluated by calculating the odds ratio (OR) and its corresponding 95% confidence interval (95% CI). The OR represents the likelihood of an event occurring in the PCS group compared to the MCS group. To further illustrate the differences between these staplers, the risk difference for anastomotic leakage and bleeding was also calculated across the included studies.

Subgroup analyses based on pathology type and year of publication were conducted to determine whether the results were homogeneous across these variables. Additionally, the Mantel–Haenszel method was used to pool the odds ratios (ORs) for the relevant outcomes, employing a random-effects model. Statistical heterogeneity between studies was assessed using the chi-square test, with *p*-value < 0.1 or *I*^2^ > 50% considered indicative of significant heterogeneity. Both Cochrane’s *Q* test and Higgins’ *I*^2^ statistic were utilized to evaluate inter-study heterogeneity. The choice between the random-effects and common-effects models was based on the level of heterogeneity observed in the included studies.

The patient populations exhibited variability in surgical techniques, demographic characteristics, and clinical conditions, reflecting a diverse, global representation. The random-effects model was selected to account for both within-study and between-study variability, providing a more conservative and generalizable estimation of the overall treatment effect. This approach acknowledges the inherent heterogeneity due to differences in surgeon expertise, geographic locations, and patient characteristics, thereby offering a more robust synthesis of evidence.

To explore potential sources of heterogeneity, subgroup analyses and sensitivity analyses were performed. Additionally, outlier detection tests were used to identify studies with extreme results, and influence analyses were conducted to assess the impact of individual studies on the overall meta-analysis.

Statistical analyses were performed using RStudio statistical software with R (version 4.3.0) with dmetar, meta, and metafor libraries. The significance level was set at *p* ≤ 0.05, and all *p*-values were two-tailed.

## Results

### Study selection and description

**Fig. 1:**
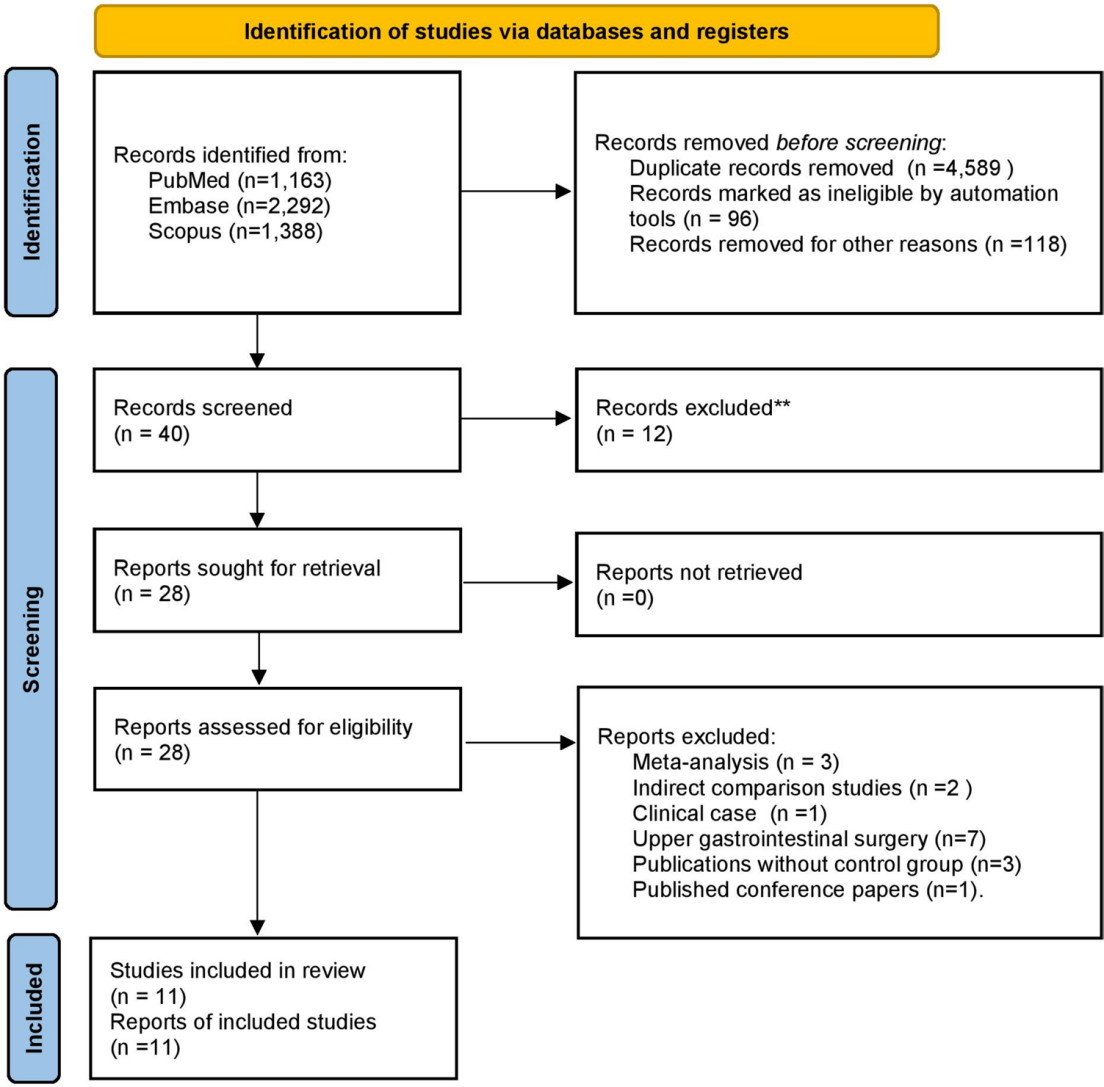
Identification of studies via databases and registers

The search in the electronic bibliographic databases yielded 4843 studies that met the research criteria (Fig. 1). Finally, 12 articles were selected for meta-analysis. One abstract publication was excluded due to duplicity as the study has been subsequently published [6, 35].

The inclusion of the two studies by Pla V et al. [6, 36] was critically evaluated by the two primary investigators. This assessment was further validated by an independent third reviewer, confirming the appropriateness of including both studies in the meta-analysis.

The characteristics of the studies included in the meta-analysis (Table 1) revealed that most were retrospective observational studies, utilizing propensity score matching for case selection in both the PCS and MCS groups (6,23,25,36–41). Six studies encompassed both benign and malignant pathologies [6, 24, 36–38], while five focused exclusively on colorectal cancer [20, 23, 25, 39, 40]. The surgical approaches varied between laparoscopic, open, and robotic techniques.

**Table 1:** Summary of main characteristics of studies included in meta-analysis

	Year	Type of study	Method	Stapler	Number of cases (after matching)		Diagnosis	Surgical approach	Anastomotic leak		Anastomotic bleed	
									MCS	PCS	MCS	PCS
Pla V et al	2021	Retrospective	PSM	ECPS	179		Mixed	Laparoscopic and open	14 (11.76%)	1 (1.67%)	-	-
					MCS: 119	PCS: 60						
Sylla P et al	2022	Retrospective	PSM	ECPS	1513		Mixed	Laparoscopic and open	93 (6.9%)	3 (1.82%)	124 (9.2%)	3 (1.82%)
					MCS: 1348	PCS: 165						
Nanishi K et al	2022	Retrospective	PSM	ECPS	271		CCR	Robotic	11 (8.87%)	10 (6.8%)	1 (0.81%)	1 (0.68%)
					MCS: 124	PCS: 147						
Gonzalez de Julian S et al	2022	Retrospective	PSM	ECPS	330		CCR	Laparoscopic and open	22 (13.33%)	8 (4.85%)	-	-
					MCS: 165	PCS: 165						
Shibutani M et al	2023	Retrospective	PSM	ECPS	126		CCR	Laparoscopic, open and robotic	9 (14.29%)	2 (3.17%)	0	0
					MCS: 63)	PCS: 63						
Vignaly A et al	2023	Retrospective	PSM	ECPS	290		Mixed	Laparoscopic and open	13 (8.97%)	8 (5.52%)	8 (5.52%)	1 (0.69%)
					MCS: 145	PCS: 145						
Matuhashi N et al	2023	Retrospective	PSM	ECPS	238		CCR	Laparoscopic	11 (7.91%)	3 (3.03%)	-	-
					MCS: 139	PCS: 99						
Chen Y et al	2024	Randomized, prospective	Clinical trial	ICS	382		CCR	¿?	4 (2.13%)	2 (1.03%)	1 (0.532%)	1 (0.513%)
					MCS: 187	PCS: 195						
Lie JJ et al	2024	Restrospective	¿?	ECPS	260		Mixed	Laparoscopic and open	3 (2%)	10 (10.8%)	-	-
					MCS: 154	PCS: 93						
Lee H et al	2024	Retrospective	PSM	ECPS + ICS	299		CCR	Laparoscopic, open and robotic	7 (4.9%)	6 (4.2%)	1 (0.7%)	2 (1.04%)
					MCS: 143	PCS: 143						
Mizumoto R et al	2024	Retrospective	PSM	ECPS	414		CCR	Laparoscopic, open and robotic	8 (3.46%)	3 (1.64%)	-	-
					MCS: 231	PCS: 183						
Pla-Martí V et al	2024	Retrospective	PSM	ECPS	330		Mixed	Laparoscopic and open	22 (13.33%)	8 (4.85%)	-	-
					MCS: 165	PCS: 165						

**Fig. 2:**
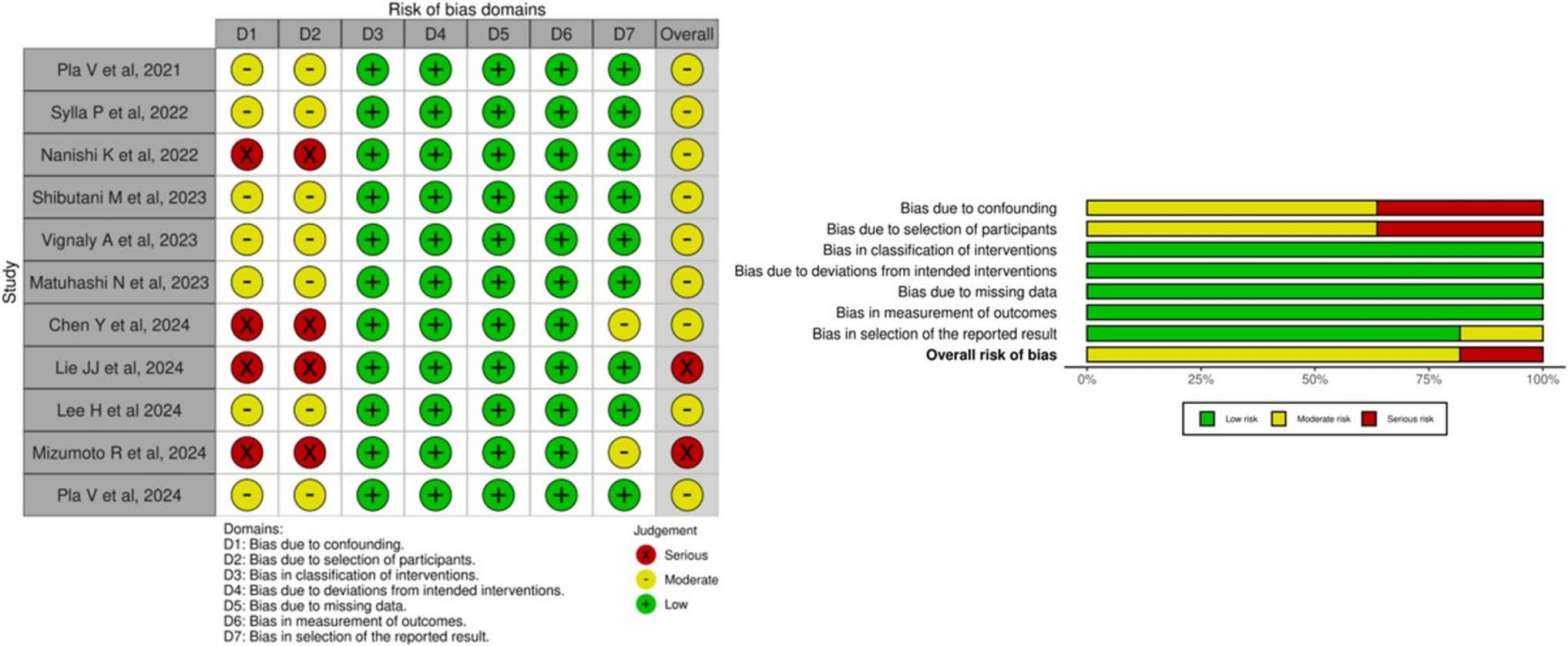
Risk of bias domains

The risk of bias was high in two studies (Fig. 2). In the remaining six studies, the risk of bias was moderate, primarily due to potential confounding factors. All other assessed domains showed a low likelihood of bias.

### Risk estimation of anastomotic leakage

#### All included studies

**Fig. 3:**
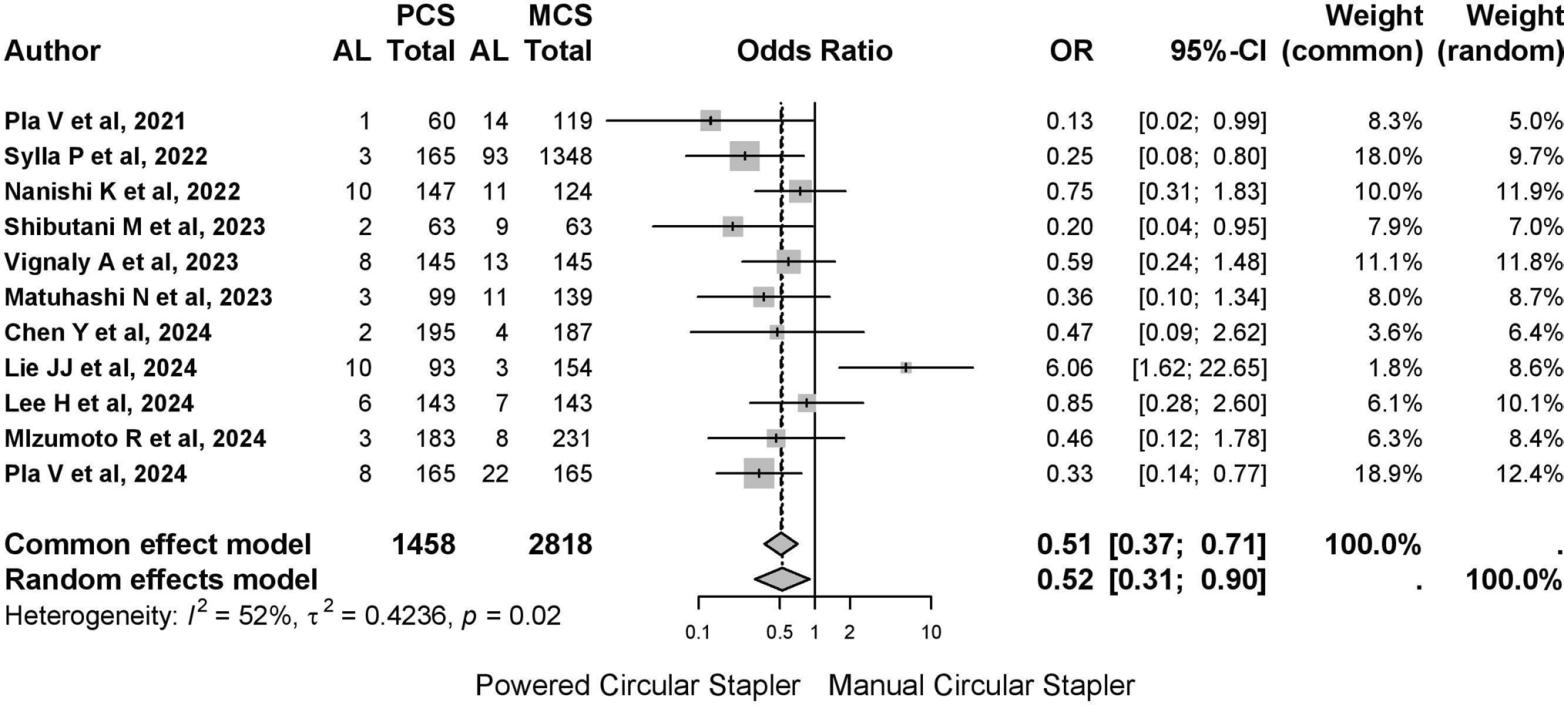
Cases of anastomotic leakage

A total of 4524 patients were included in the meta-analysis, with MCS being the most frequently used device (MCS: 3013 cases vs. PCS: 1511 cases). Among all studies, 251 cases of anastomotic leakage were reported (3.84%), affecting 56 patients in the PCS group (3.51%) and 195 patients in the MCS group (6.92%). Due to moderate heterogeneity (*I*^2^ 52.5%, 95% CI 5.7–76%; *Q* 21.04, *p* = 0.021), the random-effects model was applied. The pooled odds ratio (OR) was 0.523 (95% CI 0.306–0.898, *p* = 0.019), indicating a protective effect of PCS in reducing anastomotic leakage (Fig. 3). The risk difference (RD) between PCS and MCS was − 0.033 (95% CI − 0.06 to − 0.006), suggesting that 31 patients would need to be treated with PCS to prevent one case of leakage.

**Fig. 4:**
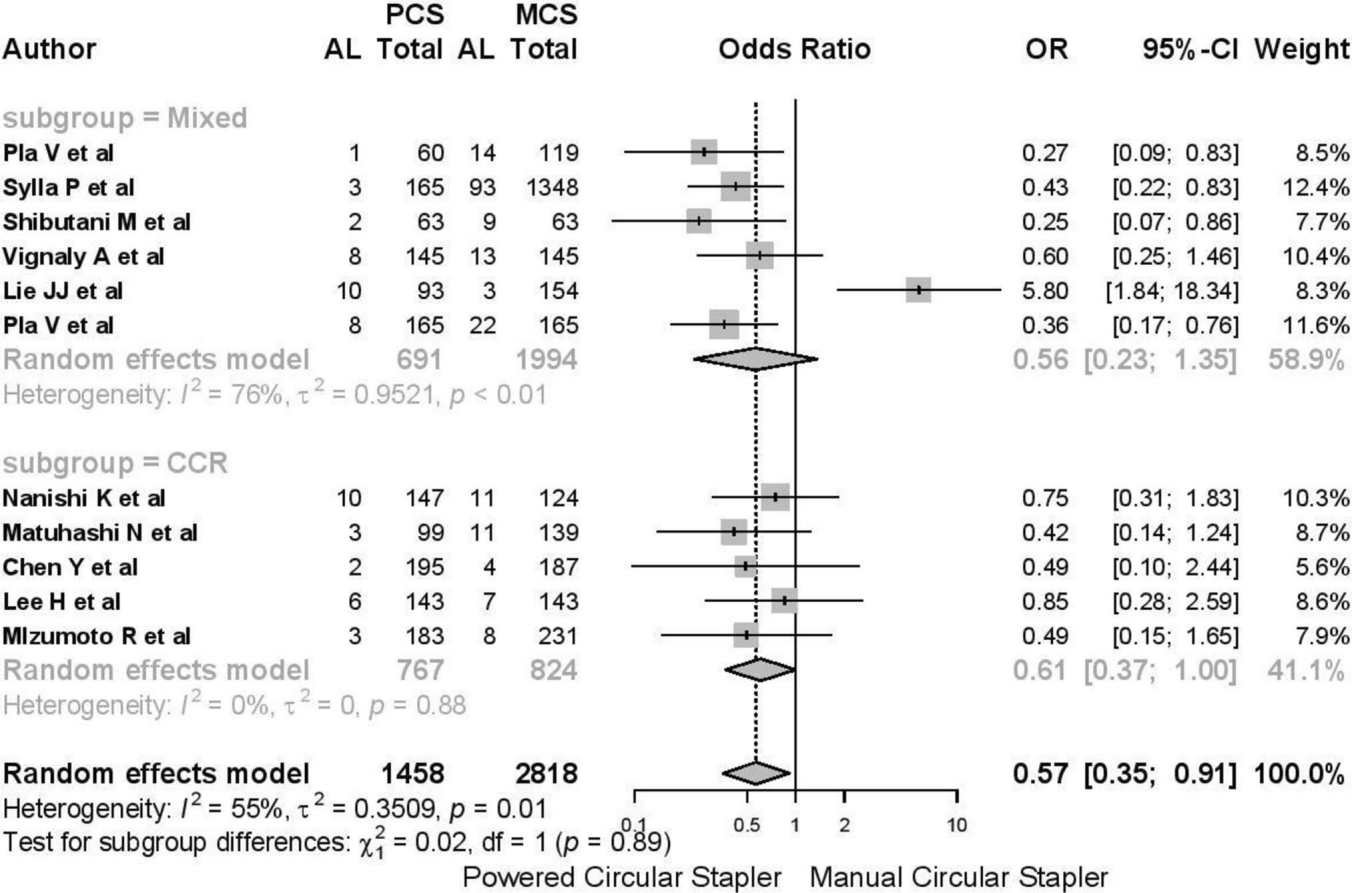
Substantial heterogeneity in studies involving mixed pathologies

Subgroup analysis revealed substantial heterogeneity in studies involving mixed pathologies (*I*^2^ 76%, *τ*^2^ 0.952, *p* < 0.01), with an OR of 0.56 (95% CI 0.23–1.35) (Fig. 4). In contrast, studies focusing exclusively on neoplasms demonstrated no heterogeneity (*I*^2^ 0%, *τ*^2^ 0, *p* = 0.88).

**Fig. 5:**
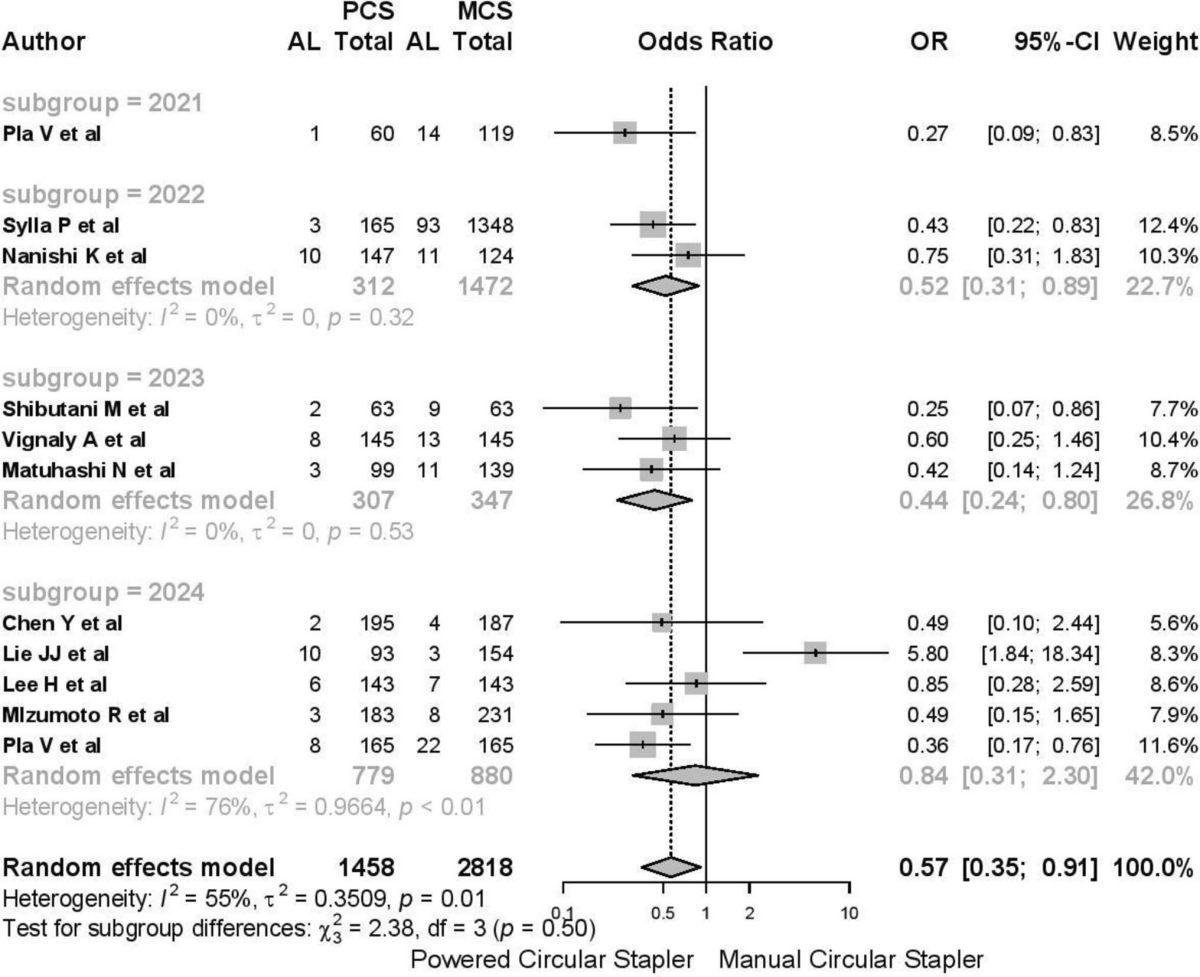
An analysis by year of publication indicated a worsening of clinical outcomes and increased heterogeneity

**Fig. 6:**
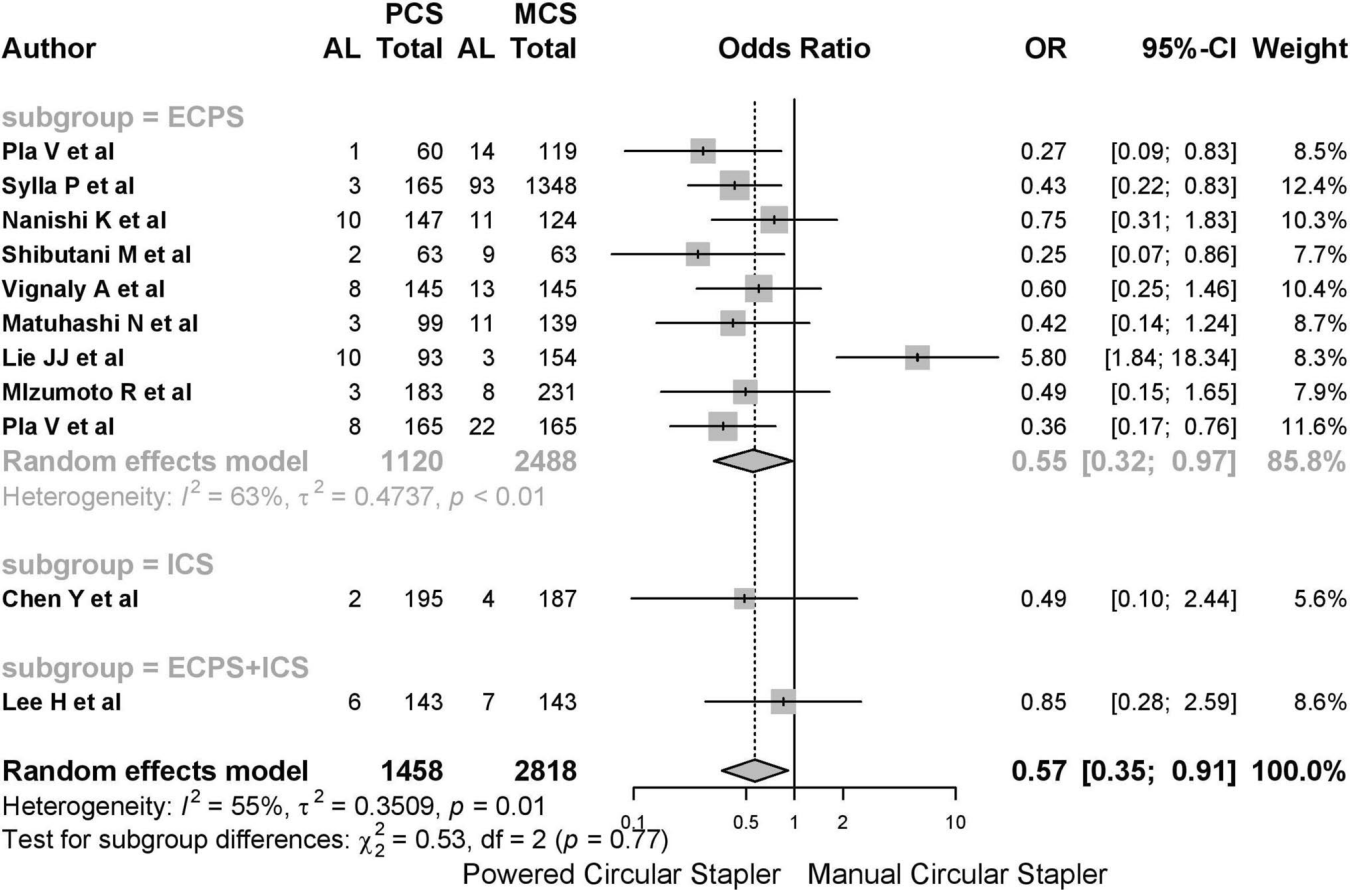
The combined EPCS + ICS group and ICS group showed higher heterogeneity and less robust results

An analysis by year of publication indicated a worsening of clinical outcomes and increased heterogeneity, particularly in studies from 2024 (Fig. 5). Regarding the type of stapler, the EPCS group demonstrated an OR of 0.52 (95% CI 0.32–0.97), while the combined EPCS + ICS group and ICS group showed higher heterogeneity and less robust results (*I*^2^ 63%, *p* < 0.01) (Fig. 6).

**Fig. 7:**
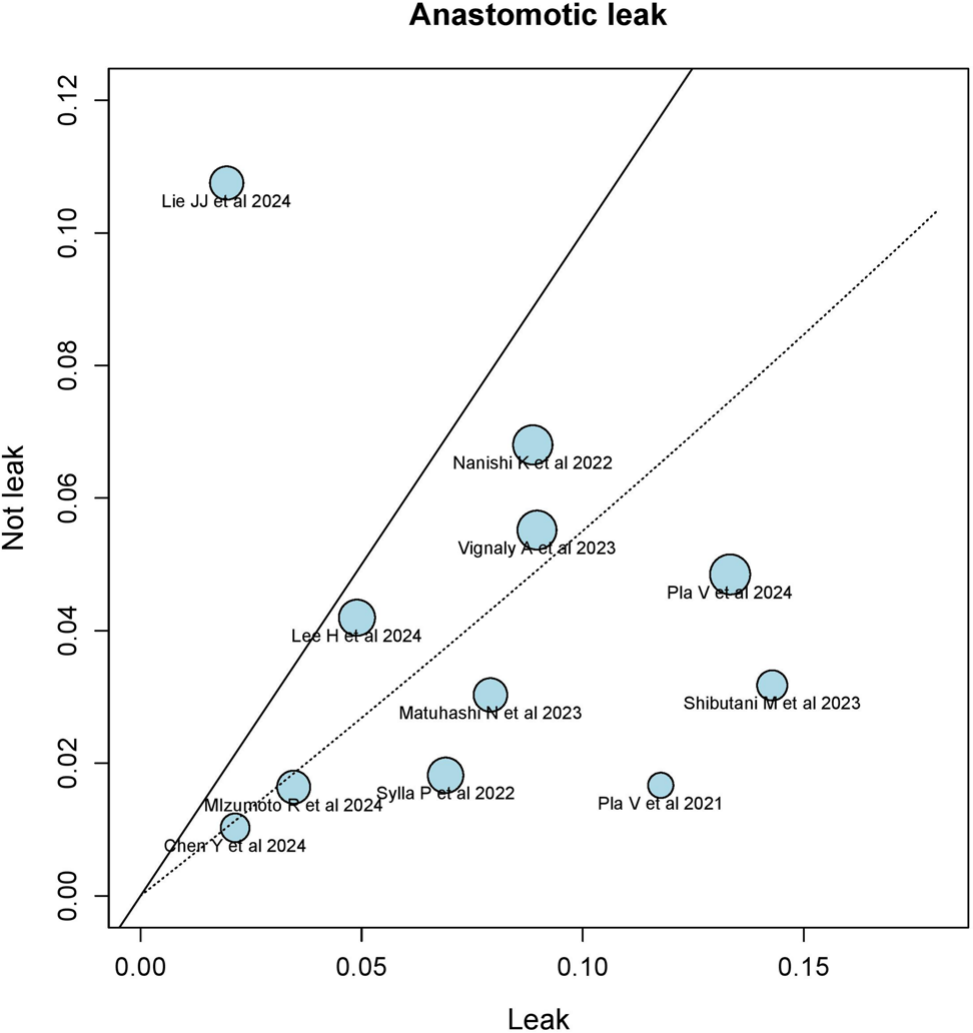
The Labbe plot suggested moderate heterogeneity

**Fig. 8:**
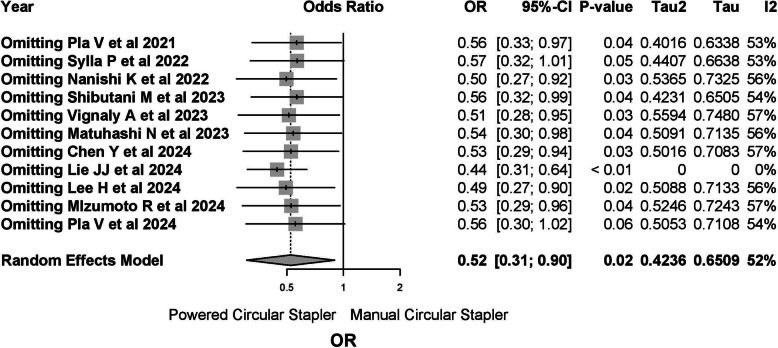
Sensitivity analysis confirmed that this study contributed significantly to the observed heterogeneity

The Labbe plot suggested moderate heterogeneity, with the findings of Lie JJ et al. being identified as an outlier (Fig. 7). Sensitivity analysis confirmed that this study contributed significantly to the observed heterogeneity (Fig. 8). Excluding Lie JJ et al. resulted in a marked reduction in heterogeneity (*I*^2^ 0%, *τ*^2^ 0%, *Q* 6.98, *p* = 0.64), allowing for the use of a common-effects model. After exclusion, the OR for leakage risk with PCS was 0.41 (95% CI 0.29–0.58), with a relative risk reduction (RR) of − 0.04 (95% CI − 0.06 to − 0.03). Twenty-five patients would need to be treated with PCS to prevent one leakage event (Supplementary Material 1, Figs. 1-2).

#### Outlier and new powered circular stapler excluded

When both Lie JJ et al. and studies involving ICS devices were excluded, heterogeneity remained low (*I*^2^ 0%, *Q* 5.48, *p* = 0.601). The common-effects model yielded an OR of 0.38 (95% CI 0.26 − 0.55), with a relative risk of −0.05 (95% CI − 0.07 to − 0.03). The number needed to treat (NNT) to prevent one leakage event was 20 (Supplementary Material 2, Figs. 1–2). Subgroup analyses by pathology type and year of publication demonstrated low heterogeneity, and no outliers were identified in the Labbe plot or influence analysis (Supplementary Material 2, Figs. 3–5).

### Estimation of the risk of anastomotic bleeding

#### All included studies

**Fig. 9:**
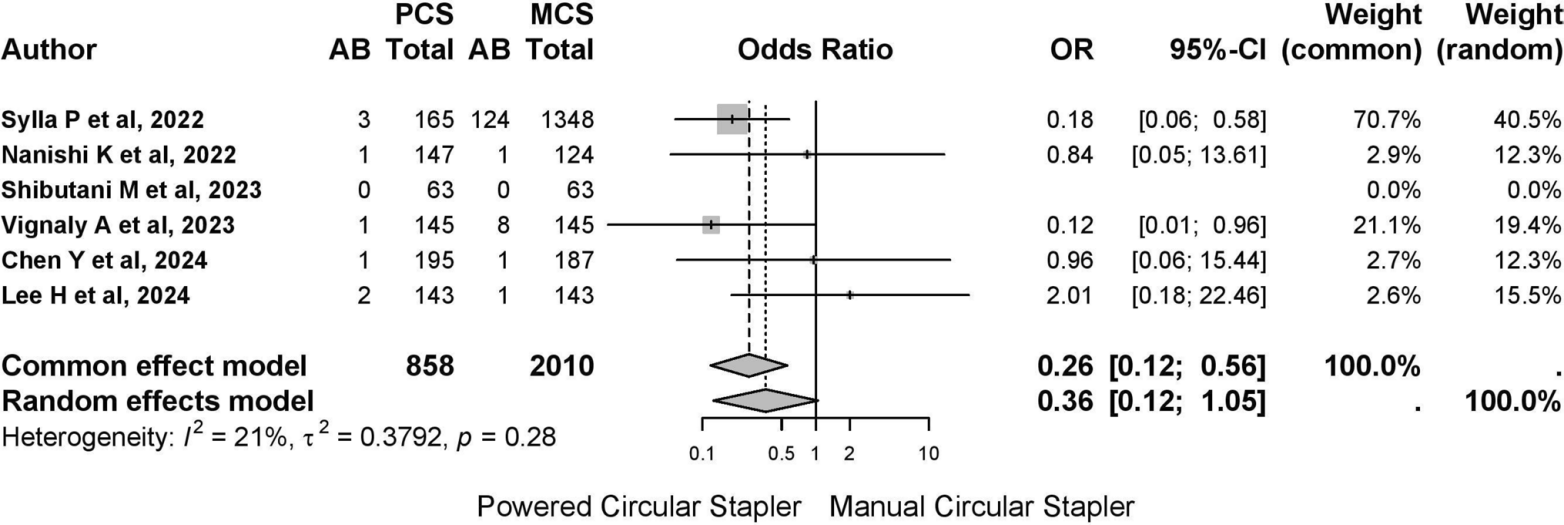
A report on anastomotic bleeding. The common-effects model was used due to low heterogeneity

Five studies reported on anastomotic bleeding, with one study reporting no cases. Among the 2868 patients included, 143 (4.99%) cases of postoperative anastomotic bleeding were observed, with 8 cases (0.923%) in the PCS group and 135 cases (6.293%) in the MCS group. The common-effects model was used due to low heterogeneity (*I*^2^ 20.8%, 95% CI 0–66.4%; *τ*^2^ 0.3792, *Q* 5.05, *p* = 0.282) (Fig. 9).

The pooled risk difference for bleeding was − 0.02 (95% CI − 0.04 to 0.01), indicating that 50 patients needed to be treated with PCS to prevent one case of bleeding.

**Fig. 10:**
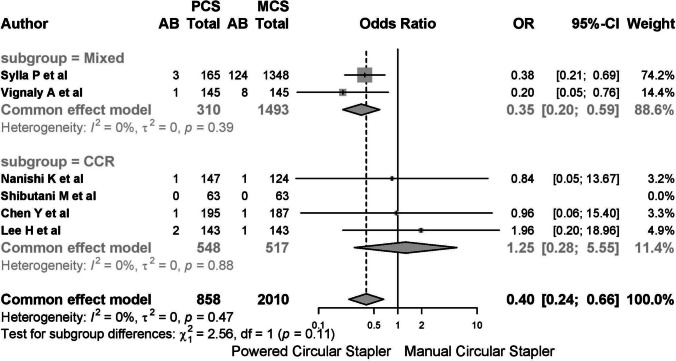
Subgroup analysis by diagnostic category

Subgroup analysis by diagnostic category (mixed vs. CRC) showed no significant differences between groups (*Q* 2.56, *p* = 0.11; *I*^2^ 0%) (Fig. 10). The OR was 0.35 (95% CI 0.20–0.59) in the mixed pathology subgroup and 1.25 (95% CI 0.28–5.55) in the CRC subgroup.

**Fig. 11:**
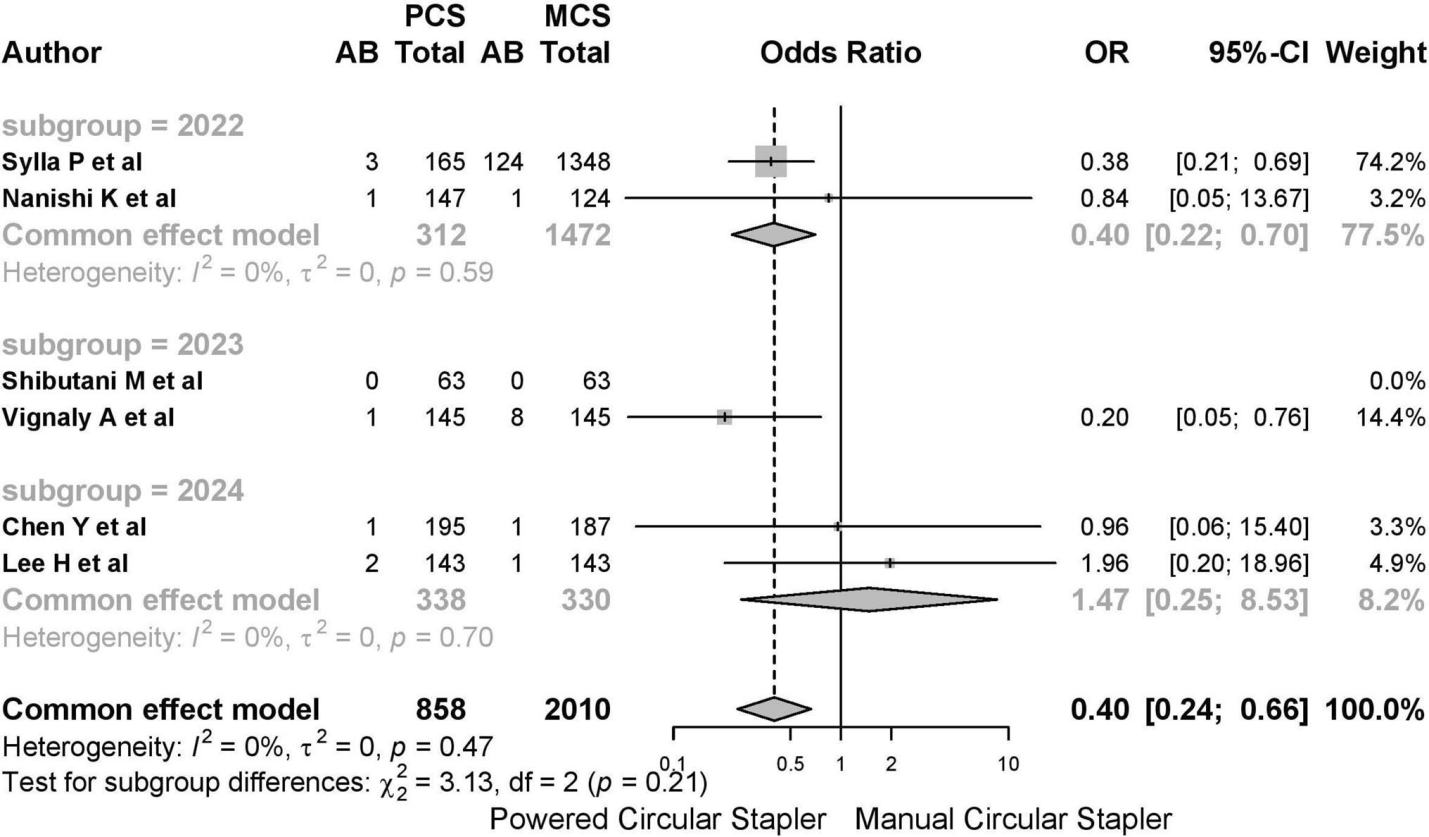
A report on anastomotic bleeding. When stratified by year, outcomes for 2024 were worse than in previous years

**Fig. 12:**
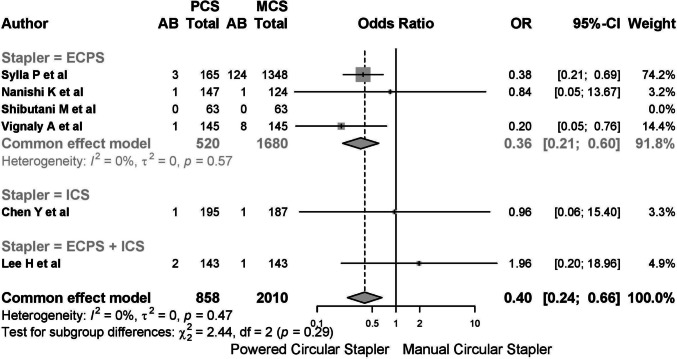
Subgroup analysis by type of stapler showed poorer outcomes in ICS and EPCS + ICS groups

When stratified by year, outcomes for 2024 were worse than in previous years (Fig. 11). Subgroup analysis by type of stapler showed poorer outcomes in ICS and EPCS + ICS groups (Fig. 12).

**Fig. 13:**
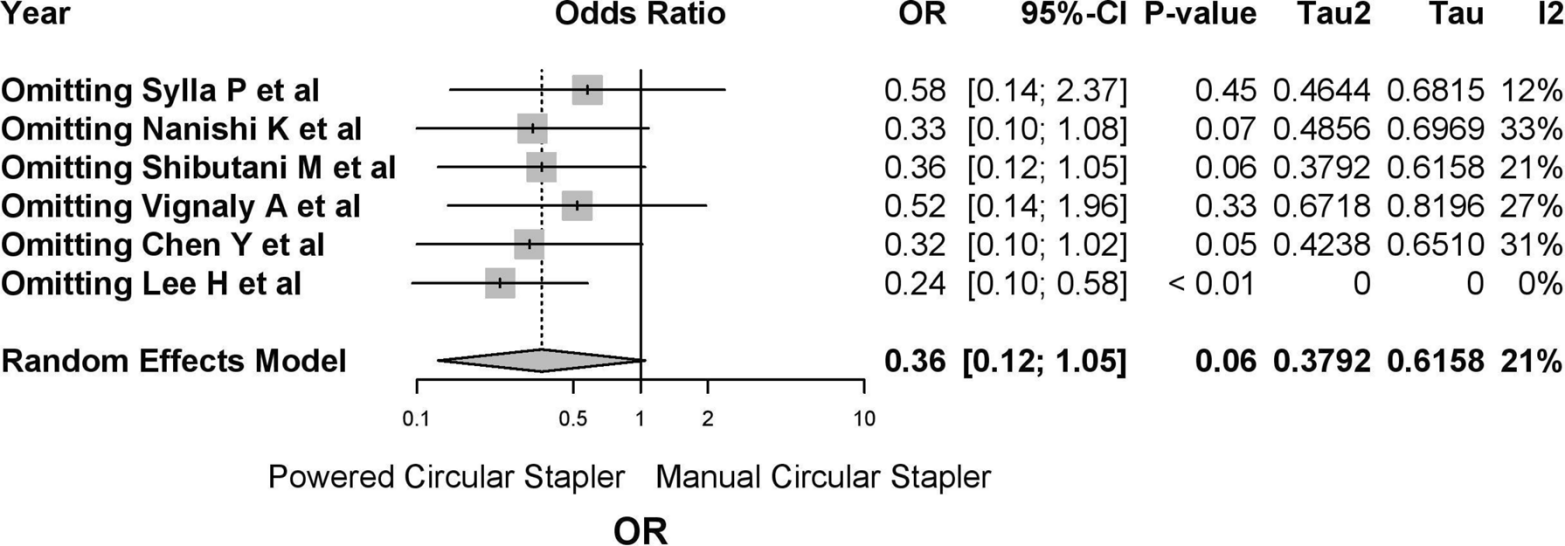
Sensitivity analysis showed minimal variation when individual studies were

Sensitivity analysis showed minimal variation when individual studies were excluded, with two studies (Sylla P et al. and Vignaly A et al.) contributing significantly to model stability. Exclusion of these studies increased heterogeneity to 31–33% and widened confidence intervals. Conversely, exclusion of Lee H et al. had minimal impact, reducing heterogeneity to 12% (Fig. 13). Influence analysis revealed moderate variability in study contributions, with no single study entirely eliminating heterogeneity.

#### New powered circular stapler excluded

Exclusion of studies involving ICS devices (Chen Y et al. and Lee H et al.) resulted in four studies with low heterogeneity (*I*^2^ 0%, *Q* 1.29, *p* = 0.526). The OR for anastomotic bleeding was 0.20 (95% CI 0.08–0.52), and the relative risk was − 0.03 (95% CI − 0.07 to − 0.01). The NNT to prevent one bleeding event was 34 (Supplementary Material 3, Figs. 1–2).

Subgroup analysis by pathology revealed an OR of 0.35 (95% CI 0.20–0.59) for the mixed pathology group and 0.36 (95% CI 0.21–0.60) for the CRC group, with no outliers observed in Labbe plots or sensitivity analyses. Influence analysis confirmed minimal heterogeneity and consistency across studies.

### Assessment of publication bias

#### Anastomotic leakage

**Fig. 14:**
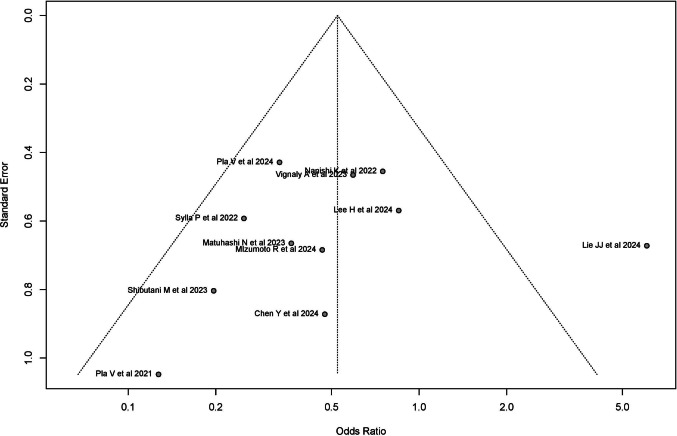
The funnel plot for the random-effects model demonstrated a symmetrical distribution of studies. Egger’s test confirmed the absence of significant asymmetry in this plot

With all studies included in the meta-analysis, the funnel plot for the random-effects model demonstrated a symmetrical distribution of studies. Egger’s test confirmed the absence of significant asymmetry in this plot (intercept: − 7.737, 95% CI − 4.27 to 2.79, *p* = 0.692) (Fig. 14).

The results of the right-skewness test (*p* = 0.666) and flatness test (*p* = 0.049) provide no evidence of significant skewness or flatness in the *p*-values of the included studies. These findings indicate a low likelihood of p-hacking practices, supporting the overall validity of the meta-analysis.

When the study by Lie JJ et al. was excluded, the funnel plot maintained symmetrical distribution of the remaining studies (Egger’s test: intercept: − 1.49, 95% CI − 3.51 to 0.33, *t* = 1.623, *p* = 0.143), and no evidence of p-hacking was observed (Right-skewness test: *p* = 0.634; flatness test: *p* = 0.833).

### Anastomotic bleeding

**Fig. 15:**
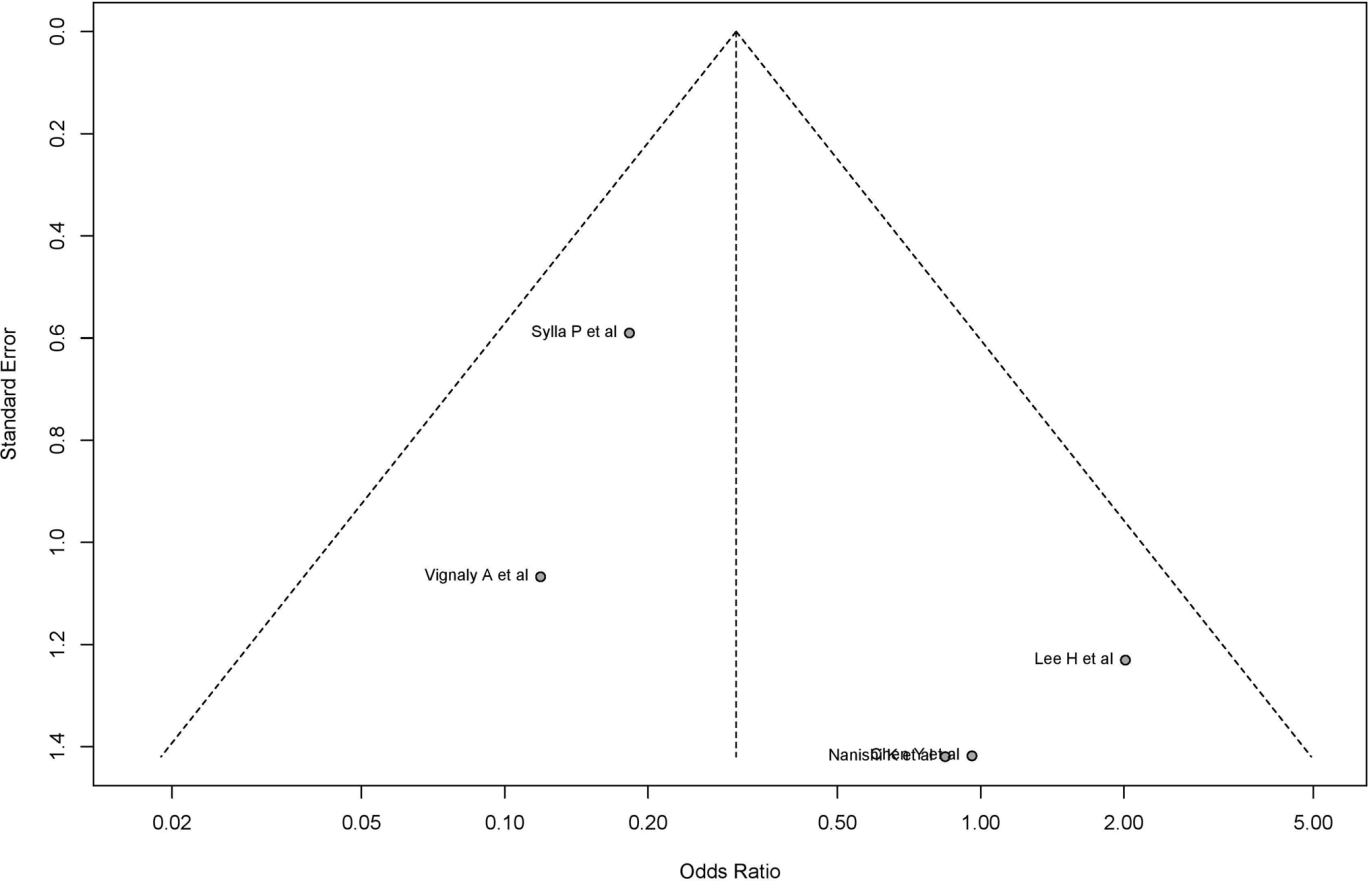
For anastomotic bleeding, the funnel plot for the random-effects model and Egger’s test showed no evidence of significant asymmetry in distribution

For anastomotic bleeding, the funnel plot for the random-effects model and Egger’s test showed no evidence of significant asymmetry in distribution (intercept: 0.988, 95% CI − 2.06 to 2.04, *p* = 0.640) (Fig. 15). Due to the limited number of cases, however, the right-skewness and flatness tests could not be performed in the anastomotic bleeding analysis. Considering only studies with EPCS, no evidence of significant asymmetry was found (intercept: 0.988, 95% CI − 2.06 to 4.04), *p*-value: 0.64).

## Grade of evidence

The GRADE table (Table 1, Supplementary Material 4) indicates that the overall quality of evidence was low when all studies were considered, for both anastomotic leaks and bleeding. However, when the outlier and studies involving mixed powered staplers or ICS were excluded, the quality of evidence improved to a moderate level.

In the analysis of anastomotic bleeding, the evidence quality also improved when only EPCS studies were included. The summary of findings after excluding outliers and studies with mixed powered staplers or ICS (Table 1, Supplementary Material 5) demonstrated that EPCS likely leads to a significant reduction in anastomotic leakage and probably results in a substantial decrease in anastomotic bleeding.

## Discussion

The growing interest in powered circular staplers is evident from the publication timeline of studies included in this meta-analysis, with almost half (45.45%) published in the first three quarters of 2024.

Conducting this meta-analysis presented several challenges, particularly due to the presence of an outlier study [24] and the introduction of a new powered stapling device [20]. The two powered circular staplers have significant differences in their design and operation.

The initial meta-analysis, which included all published studies to date, revealed significant heterogeneity (*I*^2^ = 52%), driven primarily by the Lie JJ et al. study [24] which was identified as an outlier. Excluding this study made the overall results more consistent with prior literature, and heterogeneity was significantly reduced (*I*^2^ = 0%). The outlying behaviour observed in this study may stem from methodological differences in case selection or unreported confounders.

The initial hypothesis that the results might differ between powered circular staplers was confirmed by subgroup analysis according to the type of stapler used. These differences were evident when all available studies were included, and remained with outlier study exclusion. A protective effect of EPCS against the occurrence of postoperative anastomotic leaks was observed in both cases. Due to the small number of trials using ICS, these results should be treated with caution.

A critical issue identified in the Lie JJ et al. study [24] was the significant selection bias in patient assignment to the experimental and control groups, the former consisting of patients operated on in 2021, while the latter was composed of patients from 2016. Concerns about the validity of this comparison stem from the notably worse outcomes reported for 2021 than the 2016 control group, which exhibited unexpectedly positive outcomes. Moreover, the decision to use patients from 2016 for the control group is puzzling, as more recent data (from 2018–2021 to 2022–2023) were available and would have provided a more appropriate and contemporaneous comparison. In addition, the surgical techniques used in this study, such as transanal excision of the mesorectum and total proctocolectomies with an ileoanal pouch, were different from those used in previous studies. This raises the question of whether the results of this study are truly comparable to those of previously published studies. Notably, propensity score matching to ensure comparable groups was not performed, further exacerbating the potential for bias.

The use of powered circular staplers may not be justified in experienced hands, according to Lie JJ et al. [24]. The results of this study, expected to be similar to those obtained with conventional devices rather than significantly worse, were obviously surprising [6, 15, 17, 25, 36, 37, 41–45]. The two hypotheses proposed in this study to explain these results should, however, be viewed with caution in the absence of objective evidence.

In the case of anastomotic bleeding, outcomes were superimposable to those reported in previously conducted meta-analyses [22, 46].

The variability in methodological rigor among the included studies is evident in the overall assessment of quality of evidence. While the initial analysis including all trials showed low quality evidence for both anastomotic leakage and bleeding, the level was significantly increased to moderate when excluding outliers and trials using mixed staplers or ICS. This suggests that when only the most methodologically sound studies are considered, there is moderate confidence that EPCS will result in a significant reduction in both anastomotic leakage and bleeding.

Given the potential cost differences between stapling devices, these findings may also have economic implications [6, 7], particularly in institutions where resource allocation is critical. Further cost-effectiveness analyses could help determine whether the benefits of powered staplers justify the higher costs compared to manual devices. Economic studies previously conducted on the PCS device [6, 7], in particular a cost-effectiveness study that was conducted using real-world costs [6], seem to indicate the opposite. These cost-savings are primarily due to the reduction in anastomotic leaks and associated postoperative complications, which typically increase length of hospital stay and healthcare resource utilization. Therefore, adopting advanced stapling technologies offers not only potential clinical benefits but also a clear economic advantage in high-volume centers.

The introduction of a new powered stapling device with a different design in recent studies may have influenced outcomes. Although the powered firing mechanism is popularly believed to be the primary advantage of powered stapling devices, the 3D staple design and gripping surface technology included in EPCS may play a more important role in reducing anastomotic complications. Results from studies using the newer stapler (20,23), which lacks these key features, could support this hypothesis. Despite having a similar staple design and powered firing mechanism, these studies have reported inferior outcomes, particularly in terms of anastomotic leakage. This suggests that while powered firing may contribute to the stability of the anastomotic line during the firing process, thereby decreasing the risk of small tears, the optimal tissue compression and distribution in the anastomosis achieved by 3D stapling formation and gripping surface technology could help improve perfusion and anastomotic healing conditions. These findings are consistent with our group’s hypothesis that the combination of 3D stapling and gripping surface technology are critical factors in improved anastomotic integrity [6, 22].

In this regard, future research should focus on direct comparisons between the different types of powered staplers, paying particular attention to the impact of specific design features (such as staple formation and gripping mechanisms) on anastomotic integrity and complication rates. Prospective randomized trials may help clarify these differences and guide surgeons in making evidence-based decisions in the operating room.

This study integrates all published data comparing powered circular stapling devices with manual staplers in colorectal surgery. The rigorous methodology applied highlighted qualitative differences across studies based on publication date, and the presence of studies with extreme values suggests potential performance differences between the two available powered stapling devices. By excluding studies with probable biases, this analysis provides a clearer, more accurate view of the true outcomes associated with powered circular staplers.

Due to the retrospective nature of the included studies, which may contain biases not accounted for in the analyses, the results of this study should be viewed with caution. The primary limitation of this study is the reliance mainly on retrospective studies, and that only one randomized trial was available, which may reduce the strength of the conclusions. Additionally, two studies included a newer powered stapler that differs in design from the EPCS devices, potentially introducing selection bias and affecting the comparability of outcomes within the powered stapler group.

The group is currently in the recruitment phase of an international, multicenter, randomized clinical trial with eight participating European centres (reference NCT06578065), aimed at rigorous evaluation of EPCS efficacy in reducing the incidence of anastomotic leakage and postoperative bleeding in colorectal surgery. Secondary endpoints include the impact of the device on overall complication rates, surgical efficiency and postoperative recovery time.

In conclusion, this meta-analysis demonstrates that powered circular staplers (PCS), in particular the EPCS, significantly reduce the risk of anastomotic leakage and postoperative bleeding compared with manual circular staplers (MCS). Sensitivity analyses confirmed the robustness of these findings and underscored the importance of careful study selection and standardization to minimize heterogeneity and improve reliability. These results support the use of PCS, in particular EPCS, as the preferred method in colorectal surgery. To validate these findings and address remaining uncertainties, further standardized trials are warranted.

## Supplementary Information

Below is the link to the electronic supplementary material.

Supplementary file1 (JPG 381 kb)

Supplementary file2 (DOCX 11 kb)

Supplementary file3 (JPG 340 kb)

Supplementary file4 (DOCX 12 kb)

Supplementary file5 (JPG 353 kb)

Supplementary file6 (DOCX 12 kb)

Supplementary file7 (JPG 316 kb)

Supplementary file8 (DOCX 12 kb)

Supplementary file9 (JPG 359 kb)

Supplementary file10 (DOCX 12 kb)

Supplementary file11 (JPG 387 kb)

Supplementary file12 (DOCX 12 kb)

Supplementary file13 (JPG 263 kb)

Supplementary file14 (DOCX 12 kb)

Supplementary file15 (JPG 289 kb)

Supplementary file16 (DOCX 12 kb)

Supplementary file17 (JPG 259 kb)

Supplementary file18 (DOCX 12 kb)

Supplementary file19 (DOCX 31 kb)

Supplementary file20 (DOCX 17 kb)

Supplementary file21 (DOCX 12 kb)

Supplementary file22 (DOCX 16 kb)

Supplementary file23 (DOCX 12 kb)

## Data Availability

No datasets were generated or analysed during the current study.
