# Supplementary figures and images for: Current evidence on powered versus manual circular staplers in colorectal surgery: a systematic review and meta-analysis
Source: Int J Colorectal Dis. 2025 Jan 15;40(1):13. doi: 10.1007/s00384-025-04807-y (PMC11735560; doi:10.1007/s00384-025-04807-y)

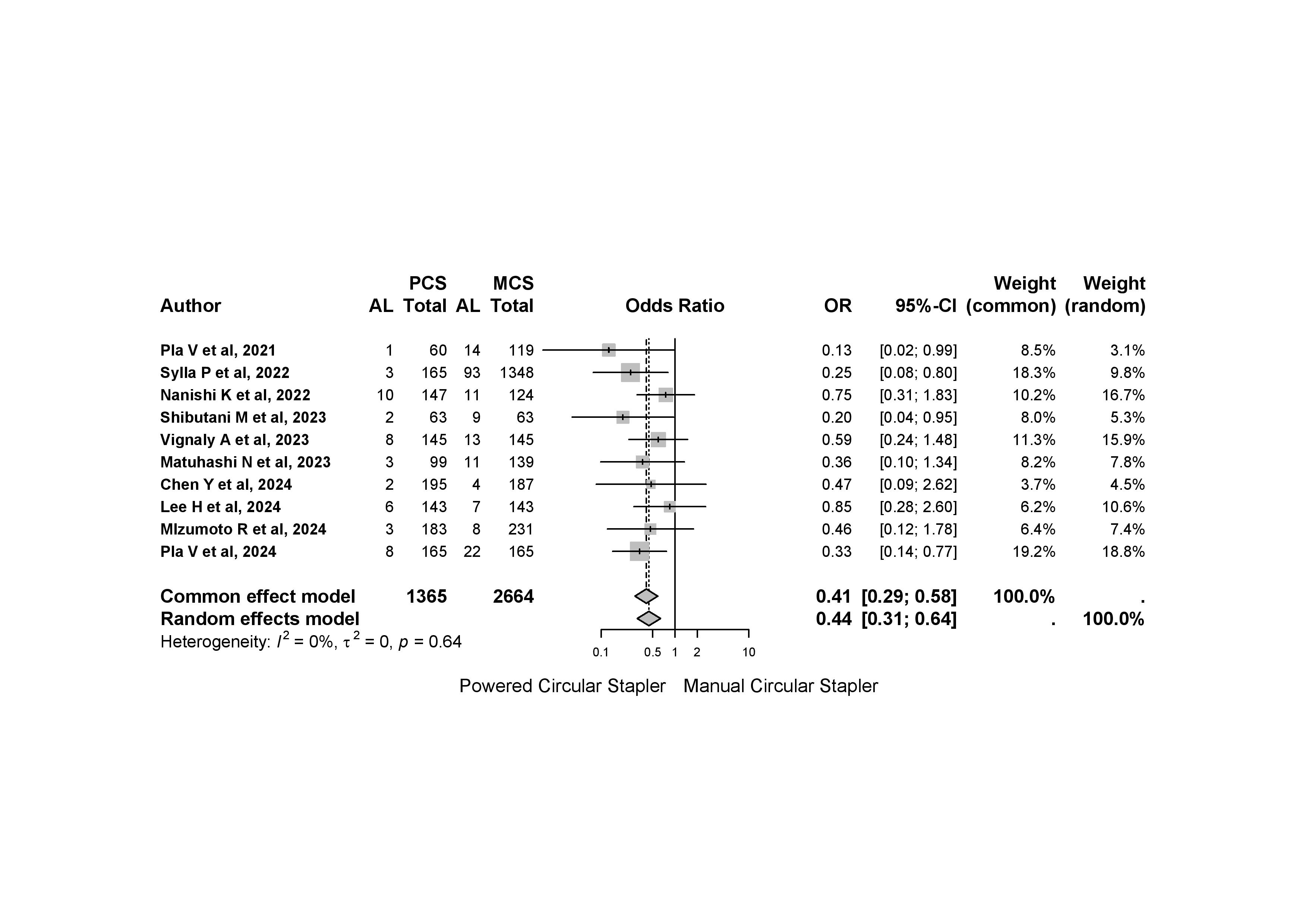

Supplement: Supplementary file 1 — Supplementary file1 (JPG 381 kb) [file 384_2025_4807_MOESM1_ESM.jpg]

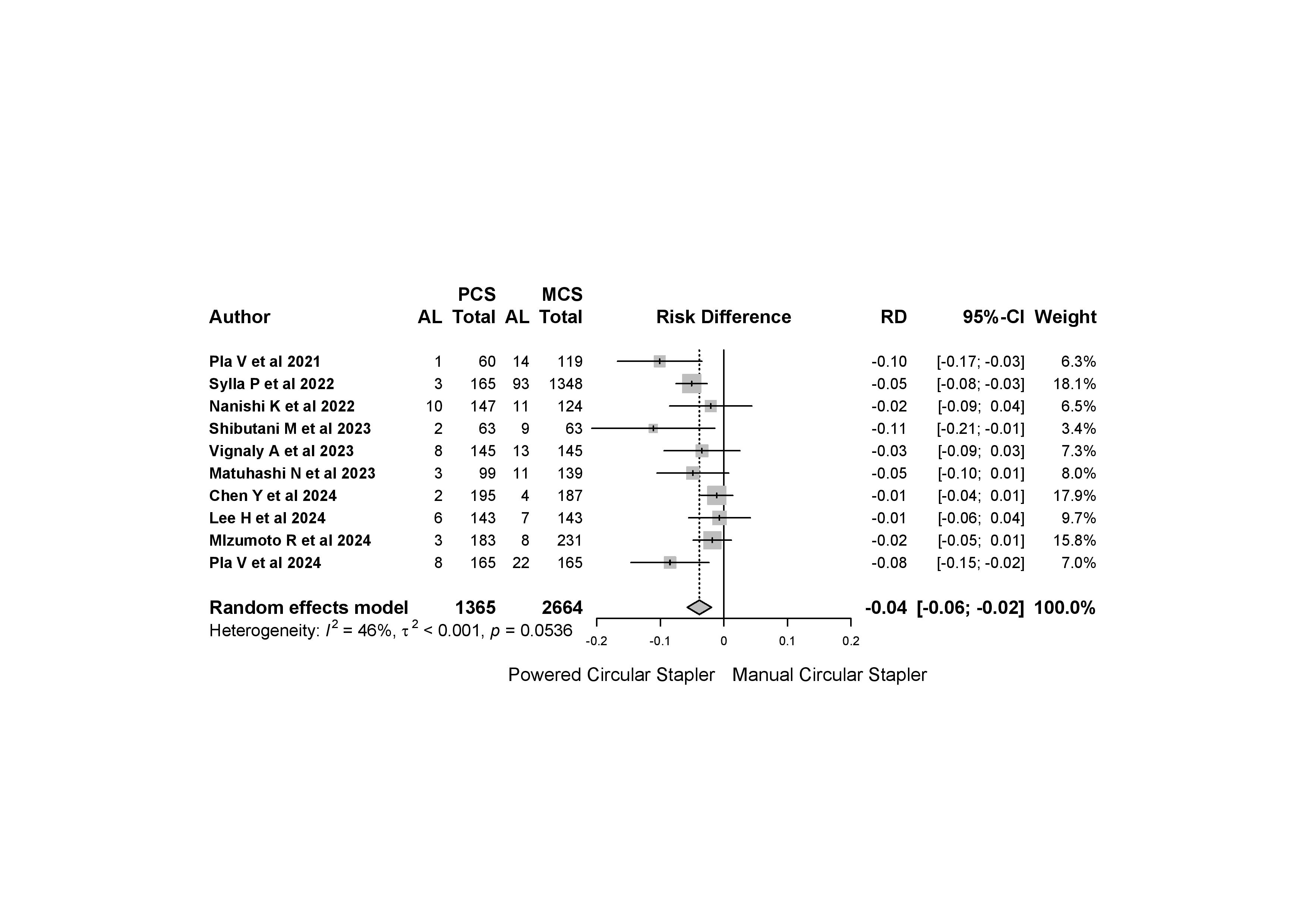

Supplement: Supplementary file 3 — Supplementary file3 (JPG 340 kb) [file 384_2025_4807_MOESM3_ESM.jpg]

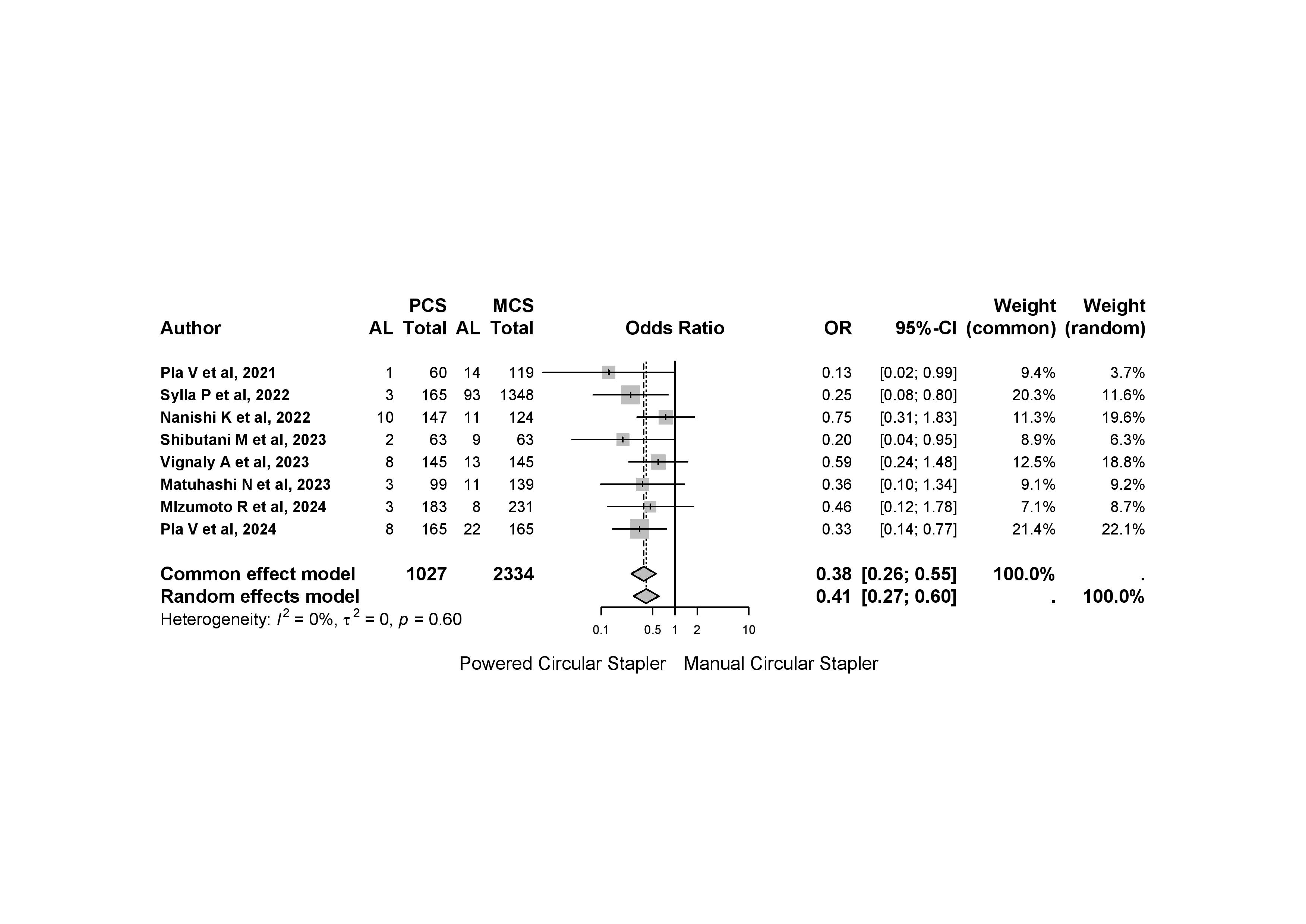

Supplement: Supplementary file 5 — Supplementary file5 (JPG 353 kb) [file 384_2025_4807_MOESM5_ESM.jpg]

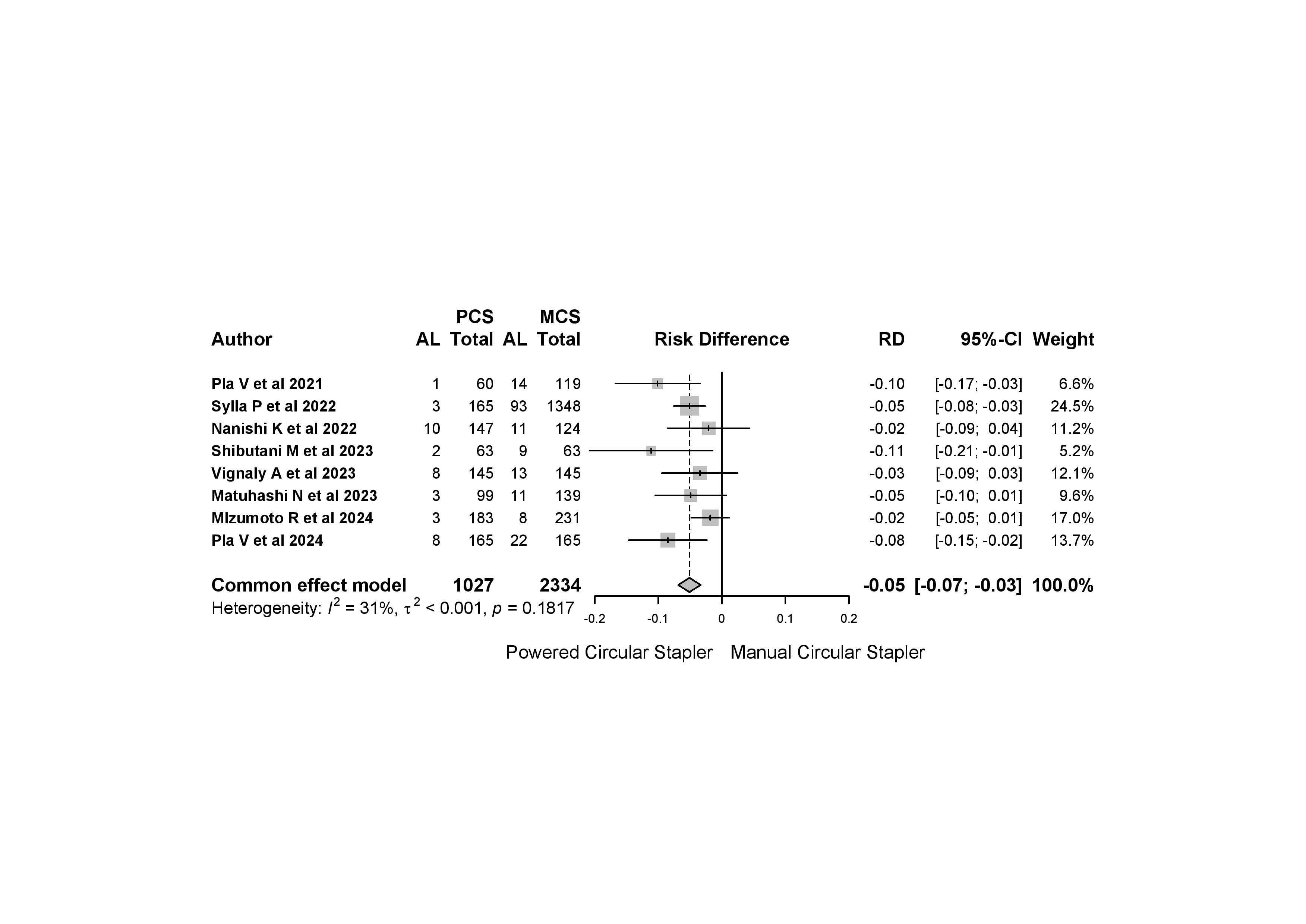

Supplement: Supplementary file 7 — Supplementary file7 (JPG 316 kb) [file 384_2025_4807_MOESM7_ESM.jpg]

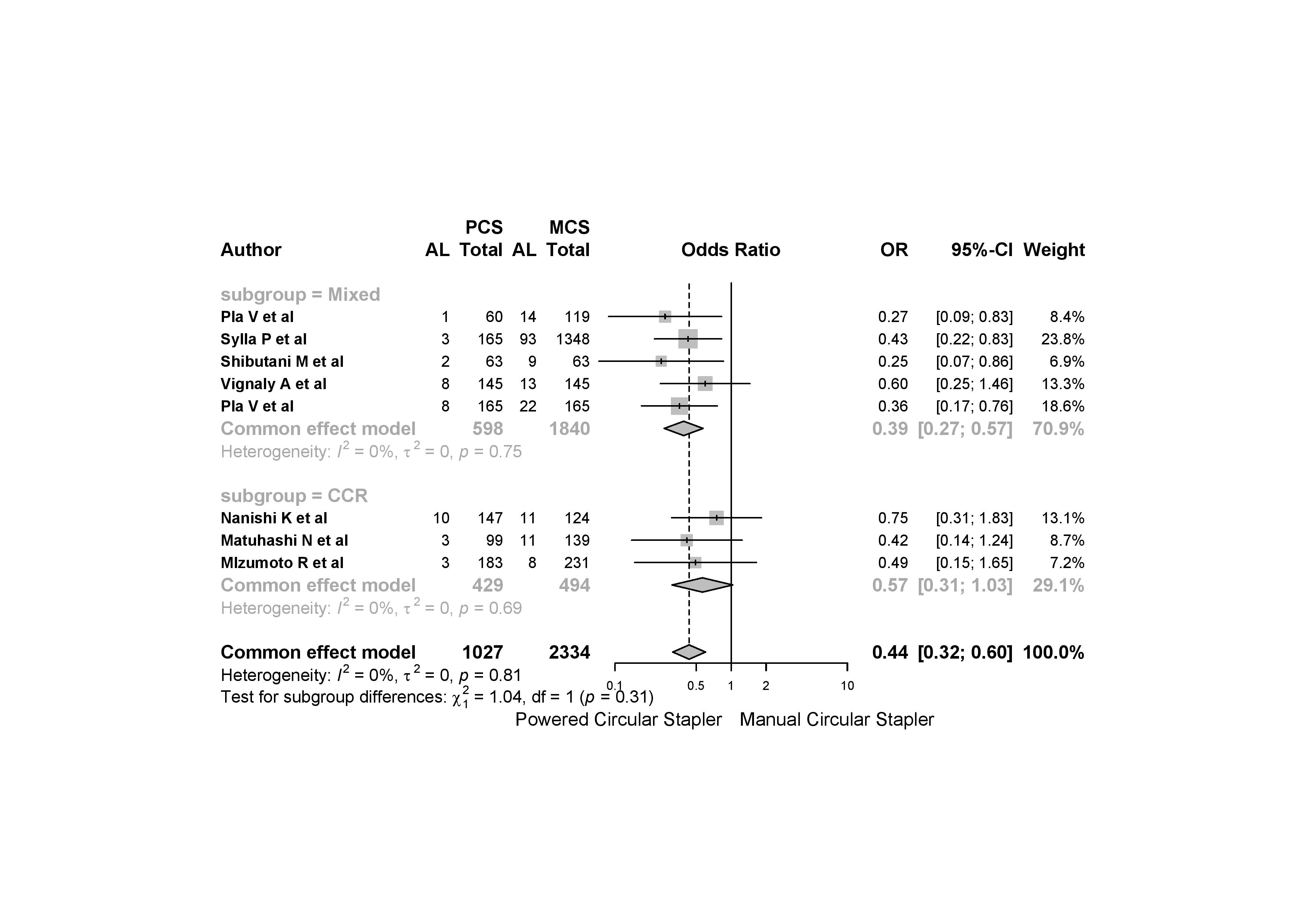

Supplement: Supplementary file 9 — Supplementary file9 (JPG 359 kb) [file 384_2025_4807_MOESM9_ESM.jpg]

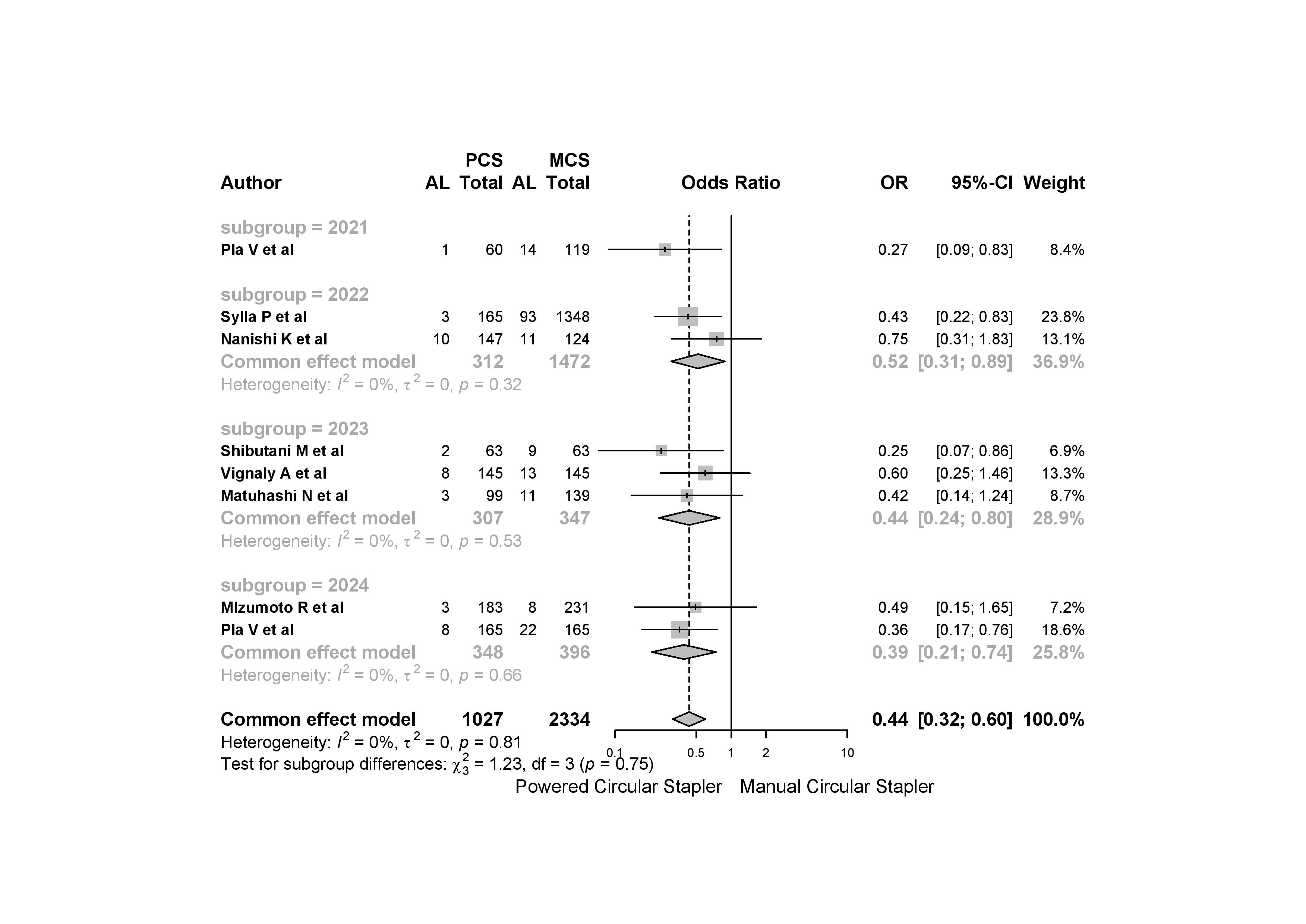

Supplement: Supplementary file 11 — Supplementary file11 (JPG 387 kb) [file 384_2025_4807_MOESM11_ESM.jpg]

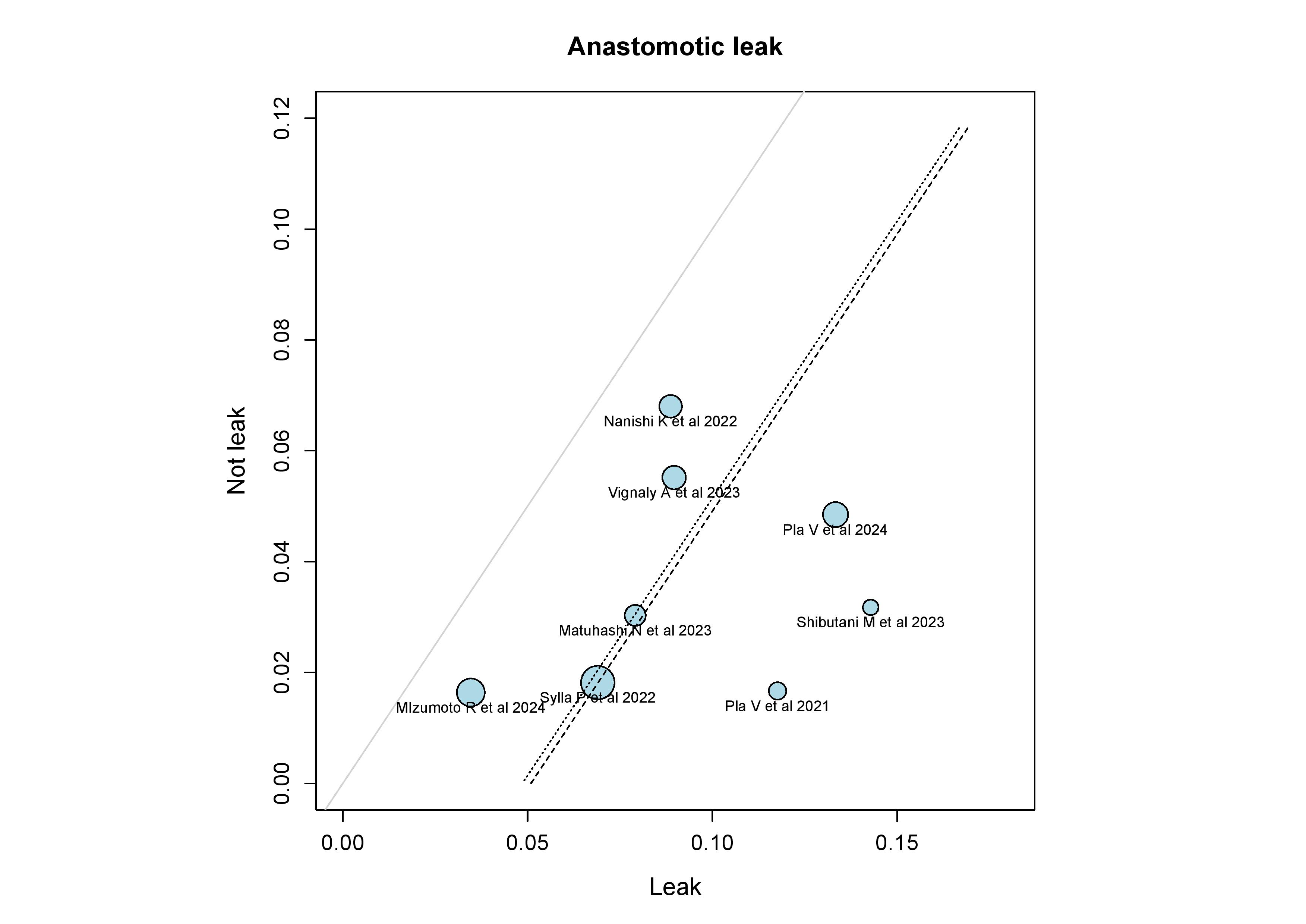

Supplement: Supplementary file 13 — Supplementary file13 (JPG 263 kb) [file 384_2025_4807_MOESM13_ESM.jpg]

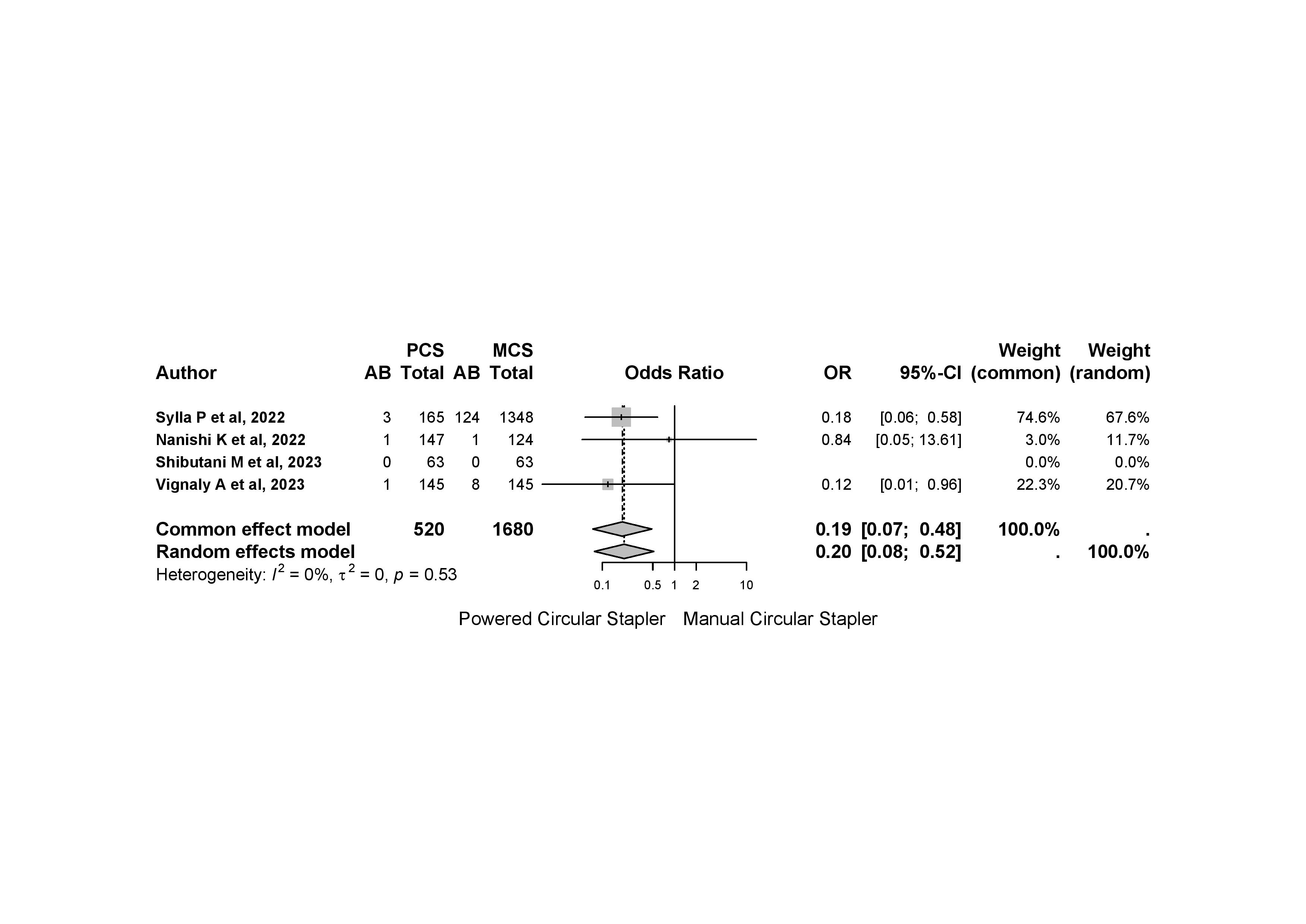

Supplement: Supplementary file 15 — Supplementary file15 (JPG 289 kb) [file 384_2025_4807_MOESM15_ESM.jpg]

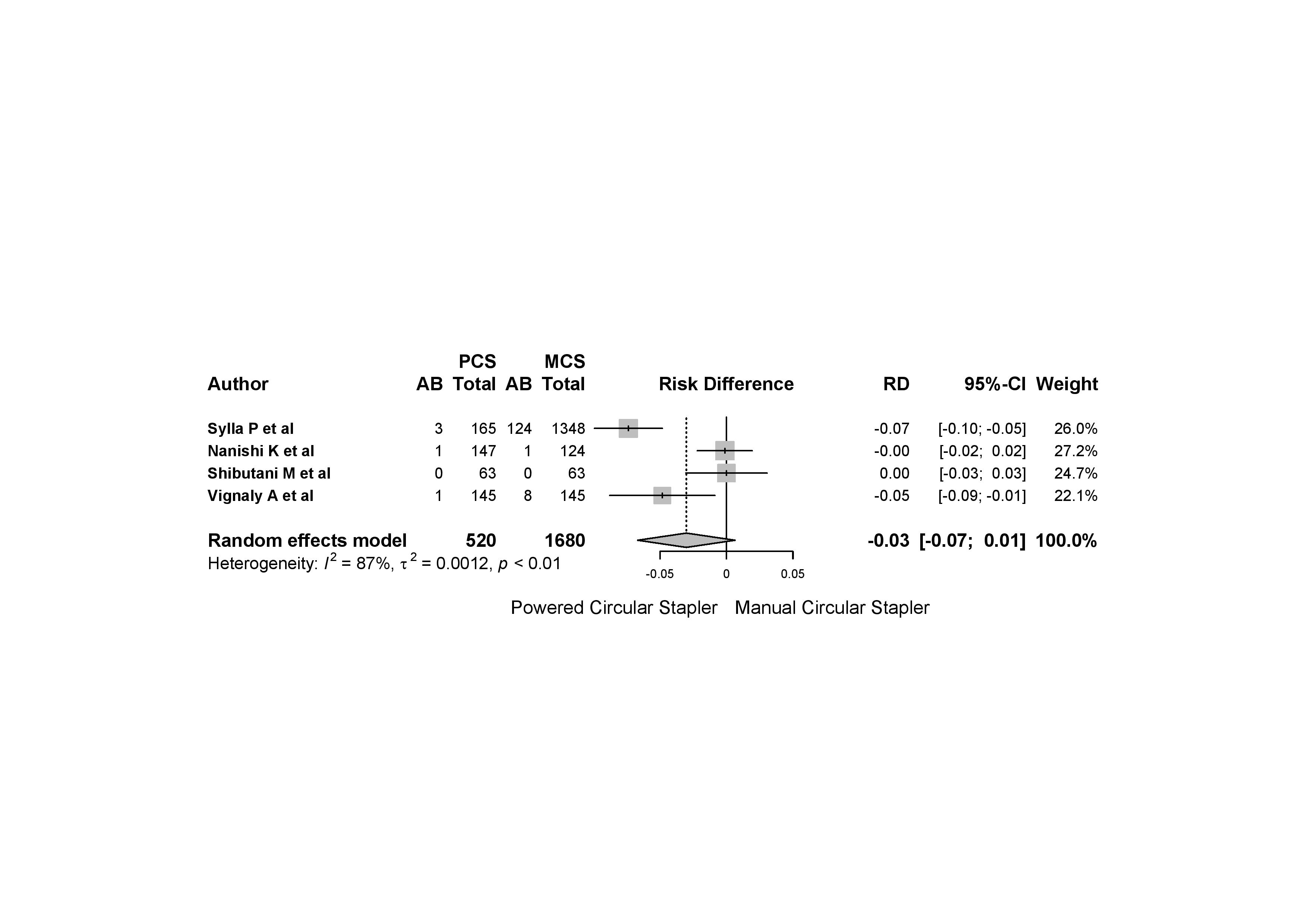

Supplement: Supplementary file 17 — Supplementary file17 (JPG 259 kb) [file 384_2025_4807_MOESM17_ESM.jpg]
