# Supplementary material for: Current evidence on powered versus manual circular staplers in colorectal surgery: a systematic review and meta-analysis
Source: Int J Colorectal Dis. 2025 Jan 15;40(1):13. doi: 10.1007/s00384-025-04807-y (PMC11735560; doi:10.1007/s00384-025-04807-y)
Supplement: Supplementary file 2 — Supplementary file2 (DOCX 11 kb) [file 384_2025_4807_MOESM2_ESM.docx]

**Figure 1.** The forest plot of the anastomotic leakage shows a low level of heterogeneity, so that the common effects model could be used.
