# Supplementary material for: Current evidence on powered versus manual circular staplers in colorectal surgery: a systematic review and meta-analysis
Source: Int J Colorectal Dis. 2025 Jan 15;40(1):13. doi: 10.1007/s00384-025-04807-y (PMC11735560; doi:10.1007/s00384-025-04807-y)
Supplement: Supplementary file 4 — Supplementary file4 (DOCX 12 kb) [file 384_2025_4807_MOESM4_ESM.docx]

**Figure 2.** The forest plot shows a moderate degree of heterogeneity in the case of the relative risk. The number of cases that needed to be treated in order to avoid a leak with a powered circular stapler was 25.
