# Supplementary material for: Current evidence on powered versus manual circular staplers in colorectal surgery: a systematic review and meta-analysis
Source: Int J Colorectal Dis. 2025 Jan 15;40(1):13. doi: 10.1007/s00384-025-04807-y (PMC11735560; doi:10.1007/s00384-025-04807-y)
Supplement: Supplementary file 14 — Supplementary file14 (DOCX 12 kb) [file 384_2025_4807_MOESM14_ESM.docx]

**Figure 5.** Forest plot of subgroup analysis by type of powered circular stapling device used did show differences in outcomes of anastomosis leaks.
