# Supplementary material for: Current evidence on powered versus manual circular staplers in colorectal surgery: a systematic review and meta-analysis
Source: Int J Colorectal Dis. 2025 Jan 15;40(1):13. doi: 10.1007/s00384-025-04807-y (PMC11735560; doi:10.1007/s00384-025-04807-y)
Supplement: Supplementary file 20 — Supplementary file20 (DOCX 17 kb) [file 384_2025_4807_MOESM20_ESM.docx]

# Supplementary Material 4.

| **Certainty assessment** | | | | | | | **№ of patients** | | **Effect** | | **Certainty** | **Importance** |
| --- | --- | --- | --- | --- | --- | --- | --- | --- | --- | --- | --- | --- |
| **№ of studies** | **Study design** | **Risk of bias** | **Inconsistency** | **Indirectness** | **Imprecision** | **Other considerations** | **Powered Circular Stapler** | **Two-row Circular Stapler** | **Relative (95% CI)** | **Absolute (95% CI)** |  |  |
| **Anastomotic Leak** | | | | | | | | | | | | |
| 11 | non-randomised studies^a^ | serious^b^ | serious^a,b,c,d,e,f^ | not serious | serious^g^ | all plausible residual confounding would reduce the demonstrated effect | 56/1458 (3.8%) | 195/2818 (6.9%) | **OR 0.52** (0.31 to 0.90) | **32 fewer per 1000** (from 47 fewer to 6 fewer) | ⨁⨁◯◯ Low^a,b,c,d,e,f,g^ | IMPORTANT |
| **Anastomotic Bleeding** | | | | | | | | | | | | |
| 6 | non-randomised studies^c^ | serious^d^ | serious^a,b,c,d,g,h^ | not serious | serious^a,b,c,d,f,g,h^ | all plausible residual confounding would reduce the demonstrated effect | 8/858 (0.9%) | 135/2010 (6.7%) | **OR 0.36** (0.12 to 1.05) | **42 fewer per 1000** (from 59 fewer to 3 more) | ⨁⨁◯◯ Low^a,b,c,d,f,g,h^ | IMPORTANT |
| **Anastomotic Leak without outliers** | | | | | | | | | | | | |
| 10 | non-randomised studies | not serious | serious^g,h^ | not serious | serious^g^ | strong association all plausible residual confounding would reduce the demonstrated effect | 49/1365 (3.6%) | 192/2664 (7.2%) | **OR 0.41** (0.29 to 0.58) | **41 fewer per 1000** (from 50 fewer to 29 fewer) | ⨁⨁◯◯ Low^g,h^ | IMPORTANT |
| **Anastomotic Leak without outliers and mixed powered circular staplers studies** | | | | | | | | | | | | |
| 8 | non-randomised studies | not serious | not serious | not serious | not serious | strong association | 38/1027 (3.7%) | 181/2334 (7.8%) | **OR 0.38** (0.26 to 0.55) | **47 fewer per 1000** (from 56 fewer to 33 fewer) | ⨁⨁⨁◯ Moderate | IMPORTANT |
| **Anastomotic bleeding without mixed circular staplers studies** | | | | | | | | | | | | |
| 4 | non-randomised studies | not serious | not serious | not serious | not serious | strong association | 5/520 (1.0%) | 133/1380 (9.6%) | **OR 0.19** (0.07 to 0.48) | **77 fewer per 1000** (from 89 fewer to 48 fewer) | ⨁⨁⨁◯ Moderate | IMPORTANT |

**CI:** confidence interval; **OR:** odds ratio

#### Explanations

a. Propensity Score Matching was performed to create comparable groups

b. One study only included rectal cancer and low colorectal anastomosis.

c. Propensity Score Marching was performed to create comparable groups

d. Ultralow anastomosis in one study

e. Restaurative proctocolectomy were included in one study

f. Selection criteria in one study were not specified

g. One study didn't show clearly outcomes

h. Different powered circular staplers were used in two studies
