# Supplementary material for: Current evidence on powered versus manual circular staplers in colorectal surgery: a systematic review and meta-analysis
Source: Int J Colorectal Dis. 2025 Jan 15;40(1):13. doi: 10.1007/s00384-025-04807-y (PMC11735560; doi:10.1007/s00384-025-04807-y)
Supplement: Supplementary file 21 — Supplementary file21 (DOCX 12 kb) [file 384_2025_4807_MOESM21_ESM.docx]

**Table 1.** GRADE table of all studies included in meta-analysis.
