# Supplementary material for: Current evidence on powered versus manual circular staplers in colorectal surgery: a systematic review and meta-analysis
Source: Int J Colorectal Dis. 2025 Jan 15;40(1):13. doi: 10.1007/s00384-025-04807-y (PMC11735560; doi:10.1007/s00384-025-04807-y)
Supplement: Supplementary file 22 — Supplementary file22 (DOCX 16 kb) [file 384_2025_4807_MOESM22_ESM.docx]

| Supplementary Material 5. | | | | | | |
| --- | --- | --- | --- | --- | --- | --- |
| **Powered Circular Stapler compared to Two-row Circular Stapler for Colorectal Anastomosis** | | | | | | |
|  | | | | | | |
| Outcome № of participants (studies) | Relative effect (95% CI) | **Anticipated absolute effects (95% CI)** | | | Certainty | What happens |
|  |  | **Two-row Circular Stapler** | **Powered Circular Stapler** | **Difference** |  |  |
| Anastomotic Leak № of participants: 4276 (11 non-randomised studies)^a^ | **OR 0.52** (0.31 to 0.90) | 6.9% | **3.7%** (2.3 to 6.3) | **3.2% fewer** (4,7 fewer to 0,6 fewer) | ⨁⨁◯◯ Low^a,b,c,d,e,f,g^ | The evidence suggests powered Circular Stapler results in a reduction in anastomotic Leak. |
| Anastomotic Bleeding (AB) № of participants: 2868 (6 non-randomised studies)^c^ | **OR 0.36** (0.12 to 1.05) | 6.7% | **2.5%** (0.9 to 7) | **4.2% fewer** (5,9 fewer to 0,3 more) | ⨁⨁◯◯ Low^a,b,c,d,f,g,h^ | The evidence suggests powered Circular Stapler results in a reduction in anastomotic Bleeding. |
| Anastomotic Leak without outliers (AL) № of participants: 4029 (10 non-randomised studies) | **OR 0.41** (0.29 to 0.58) | 7.2% | **3.1%** (2.2 to 4.3) | **4.1% fewer** (5 fewer to 2,9 fewer) | ⨁⨁◯◯ Low^g,h^ | Powered Circular Stapler may result in a reduction in anastomotic Leak without outliers. |
| Anastomotic Leak without outliers and mixed powered circular staplers studies № of participants: 3361 (8 non-randomised studies) | **OR 0.38** (0.26 to 0.55) | 7.8% | **3.1%** (2.1 to 4.4) | **4.7% fewer** (5,6 fewer to 3,3 fewer) | ⨁⨁⨁◯ Moderate | Powered Circular Stapler likely results in a large reduction in anastomotic Leak without outliers and mixed powered circular staplers studies. |
| Anastomotic bleeding without mixed circular staplers studies № of participants: 1900 (4 non-randomised studies) | **OR 0.19** (0.07 to 0.48) | 9.6% | **2.0%** (0.7 to 4.9) | **7.7% fewer** (8,9 fewer to 4,8 fewer) | ⨁⨁⨁◯ Moderate | Powered Circular Stapler probably results in a large reduction in anastomotic bleeding without mixed circular staplers studies. |
| ***The risk in the intervention group** (and its 95% confidence interval) is based on the assumed risk in the comparison group and the **relative effect** of the intervention (and its 95% CI).  **CI:** confidence interval; **OR:** odds ratio | | | | | | |
| **GRADE Working Group grades of evidence** **High certainty:** we are very confident that the true effect lies close to that of the estimate of the effect. **Moderate certainty:** we are moderately confident in the effect estimate: the true effect is likely to be close to the estimate of the effect, but there is a possibility that it is substantially different. **Low certainty:** our confidence in the effect estimate is limited: the true effect may be substantially different from the estimate of the effect. **Very low certainty:** we have very little confidence in the effect estimate: the true effect is likely to be substantially different from the estimate of effect. | | | | | | |

#### Explanations

a. Propensity Score Matching was performed to create comparable groups

b. One study only included rectal cancer and low colorectal anastomosis.

c. Propensity Score Marching was performed to create comparable groups

d. Ultralow anastomosis in one study

e. Restaurative proctocolectomy were included in one study

f. Selection criteria in one study were not specified

g. One study didn't show clearly outcomes

h. Different powered circular staplers were used in two studies
